# Detection Technologies for Reactive Oxygen Species: Fluorescence and Electrochemical Methods and Their Applications

**DOI:** 10.3390/bios11020030

**Published:** 2021-01-24

**Authors:** Surachet Duanghathaipornsuk, Eveline J. Farrell, Ana C. Alba-Rubio, Piotr Zelenay, Dong-Shik Kim

**Affiliations:** 1 Department of Chemical Engineering, The University of Toledo, Toledo, OH 43606, USA; Surachet.Duanghathaipornsuk@rockets.utoledo.edu (S.D.); Eveline.Farrell@rockets.utoledo.edu (E.J.F.); Ana.AlbaRubio@UToledo.edu (A.C.A.-R.); 2 Materials Physics and Applications Division, Los Alamos National Laboratory, Los Alamos, NM 87545, USA; zelenay@lanl.gov

**Keywords:** reactive oxygen species, fluorescence sensor, electrochemical sensor, in vivo detection, oxidative-stress-related disease

## Abstract

Reactive oxygen species (ROS) have been found in plants, mammals, and natural environmental processes. The presence of ROS in mammals has been linked to the development of severe diseases, such as diabetes, cancer, tumors, and several neurodegenerative conditions. The most common ROS involved in human health are superoxide ${(O}_{2}^{•-})$, hydrogen peroxide (H_2_O_2_), and hydroxyl radicals (•OH). Organic and inorganic molecules have been integrated with various methods to detect and monitor ROS for understanding the effect of their presence and concentration on diseases caused by oxidative stress. Among several techniques, fluorescence and electrochemical methods have been recently developed and employed for the detection of ROS. This literature review intends to critically discuss the development of these techniques to date, as well as their application for in vitro and in vivo ROS detection regarding free-radical-related diseases. Moreover, important insights into and further steps for using fluorescence and electrochemical methods in the detection of ROS are presented.

## 1. Introduction

The oxygen derivatives produced from complete or incomplete oxidation are considered reactive oxygen species (ROS). The definition of ROS not only applies to free-radical molecules, such as superoxide ${(O}_{2}^{•-})$, hydroperoxyl (HO_2_), hydroxyl (•OH), peroxy (ROO•), and alkoxy (RO•) radicals, but also hydrogen peroxide (H_2_O_2_) and singlet oxygen (^1^O_2_) are included as nonradical reactive molecules. The most commonly found ROS are $O_{2}^{•-}$, H_2_O_2_, and •OH, which are mainly generated in plants [1], mammals [2,3], and natural environmental processes [4]. ROS can be found in natural water in the environment [5], cigarette smoke [6], and air pollutants [7], in which photolysis and electron transfer are the main reactions for their generation [8]. Moreover, it was speculated by Boursiac et al. that ROS play a crucial role as a second messenger in the regulation of water transport in plant roots [9] and are vital in stress signaling and refutation of acclimation responses in plants under stress conditions [10]. ROS are also produced in mammals as normal metabolic species and can provide either benefits or drawbacks, depending on their concentration. Mitochondria [11], the family of nicotinamide adenine dinucleotide phosphate (NADPH) oxidases (NOXs) [12], and oxidative burst from respiratory proteins such as hemoglobin [13], are the production sites for ROS in mammals. For example, ROS are produced via a one-electron reduction of O_2_ in mitochondria to generate water, in which a small amount of $O_{2}^{•-}$ can be produced as a result of the reduction process. Superoxide dismutases (SODs) in the intermembrane of mitochondria will then transform $O_{2}^{•-}$ into either a less reactive ROS such as H_2_O_2_ [14] or the strongest oxidant species (i.e., •OH) [15].

At low concentrations, ROS provide constructive functions in normal cell metabolism under physiological conditions. ROS benefit living cells by acting as a defense mechanism against bacteria, chemical messengers for receptor-mediated signaling pathways [16], and transcriptional activation [17,18]. However, ROS are commonly considered harmful species because they may contribute to oxidative stress, leading to tissue damage and contributing to disease when the cellular antioxidant defense systems cannot maintain the presence of ROS at low concentrations. Cancer [19], skin aging [20], diabetes [21], heart disease [22], tumors [23], and several neurodegenerative diseases [24,25,26] may be the result of cell damage from a high concentration of ROS, which can be referred to as oxidative-stress-related diseases. The detection of ROS has recently drawn much more attention in academic, medical, and industrial setups. For example, in biology, the ability to achieve real-time detection of ROS can catalyze a deeper understanding of the functions of ROS in plants, bacteria [27], or even the mitochondria of mammalian cells. Furthermore, early detection of changes in the concentration of ROS seems essential for pathological studies, disease diagnosis, and health screening in the medical field [28]. The development of proton exchange membrane fuel cells (PEMFCs) in the automotive industry is another field that could benefit from the detection of ROS, as these are well known for degrading the membrane, thus reducing the lifetime of fuel cells [29].

As innovation continues, there are several limitations that need to be considered for ROS detection development. One of the main challenges is the distinctive intrinsic properties of different ROS, such as lifetime, diffusion rate, and their different generation sources, which could lead to inaccurate and inconsistent measurements. Moreover, their low and unstable concentrations at these generation sources can cause several techniques to be inappropriate for ROS detection, especially in living cells. Researchers have developed certain techniques, such as electron spin resonance (ESR) [30,31], mass spectrometry (MS) [32], spectrophotometry [33,34], high-performance liquid chromatography (HPLC) [35,36], fluorescence spectroscopy [37,38], and electrochemical techniques [39,40]. However, there is no universal method that can provide direct and consistent detection of ROS at the generation source without sampling and transferring procedures or sample pretreatment. Recently, different techniques for ROS detection in living cells with easy procedures, high sensitivity, and selectivity have arisen, such as the fluorescence method and electrochemical techniques.

In this review, we focus not only on the recent trends of the fluorescence method and electrochemical techniques for measuring $O_{2}^{•-}$, H_2_O_2_, and •OH in cellular systems, but also on the capacity of these techniques for diagnosing diseases related to high concentrations of ROS.

## 2. Superoxide Radical ($O_{2}^{•-})$ Detection

### 2.1. Introduction to Superoxide Radical ($O_{2}^{•-}$) Detection

The superoxide radical ($O_{2}^{•-})$ is generated in mitochondria by the incomplete redox reaction of oxygen molecules or during the production of adenosine triphosphate (ATP). Studies on the role of $O_{2}^{•-}$ in mammalian cells point out that $O_{2}^{•-}$ plays a crucial role in various physiological functions due to its involvement as a secondary messenger in signaling pathways [41] and in the regulation of gene expression [42]. Though $O_{2}^{•-}$ is produced during normal metabolic processes and is required to maintain physiological functions, its concentration is controlled by the balance between the production by enzymes and the elimination by antioxidants. Excessive $O_{2}^{•-}$ concentrations are the result of a weakened antioxidant system and/or mitochondrial malfunction, which can lead to the destruction of adjacent proteins around the generation source of $O_{2}^{•-}$ and the development of a state of oxidative stress. The oxidative stress caused by $O_{2}^{•-}$ is due to a combination of cellular damage and stress-responsive signaling. $O_{2}^{•-}$ does not contribute to randomized protein damage, but, instead, affects specific sensitive proteins. These specific proteins respond to the accumulation of $O_{2}^{•-}$ by either adapting to the higher concentration [43] or by initiating cell death [44,45]. The severe oxidative damage resulting from oxidative stress could contribute to the development of different diseases, such as atherosclerosis, autism, and Parkinson’s and Alzheimer’s diseases [46]. As the concentration of $O_{2}^{•-}$ can be used as a potential biomarker for early diagnosis, precise detection of $O_{2}^{•-}$ is necessary for investigating and understanding the development of oxidative-stress-related diseases.

While low concentrations of intracellular $O_{2}^{•-}$ (10–100 nM) are considered normal, these can increase by three or four orders of magnitude to 0.1 mM under environmental stress conditions or during illness, which highlights the importance of developing technologies with ultralow detection limits in order to assess different sources of oxidative stress. For example, by monitoring released $O_{2}^{•-}$ from human malignant melanoma cells under drug stimulation, there have been great improvement in the real-time detection and sensitivity of the measurements [47]. As previously stated, detection of $O_{2}^{•-}$ is not straightforward due to its high reactivity and short half-life. Therefore, real-time detection techniques are critical in order to accurately report the biological impact of the presence of $O_{2}^{•-}$ at elevated concentrations, which are related to the body’s immune response.

Although conventional methods, such as electron spin resonance (ESR), mass spectrometry (MS), high-performance liquid chromatography (HPLC), etc., are commonly used for the detection of $O_{2}^{•-}$, a critical disadvantage is that most of these techniques cannot be used to detect at the generation source (in situ detection). Moreover, most of them are only suitable for detection at high concentrations, while the ability to measure at low concentration levels requires additional sensitivity. Among the different available techniques, fluorescence and electrochemical methods have shown to be the most promising for $O_{2}^{•-}$ detection due to their high sensitivity and ability to measure at the generation source. The main advantages of both techniques are the ability to penetrate through cells, mitochondria, or targeted sites [48,49,50,51] in order to gather information about the oxidative state of those cellular compartments for the detection of $O_{2}^{•-}$. The use of fluorescent probes is considered an effective method due to the high spatial and temporal resolution [52,53], decreased invasiveness, and excellent sensitivity and selectivity with a rapid response [54,55,56]. Moreover, the fluorescence method has been developed to detect $O_{2}^{•-}$ in living cells (real-time detection). However, the degree of accuracy, cell permeability, and intracellular stability, while maintaining low toxicity, still requires further improvement. Thus, in the first part of this section, we will mainly focus on $O_{2}^{•-}$ detection in disease-related tissues and organs using the fluorescence method.

### 2.2. Fluorescence Method for Superoxide Radical ($O_{2}^{•-}$) Detection

**Figure 1:**
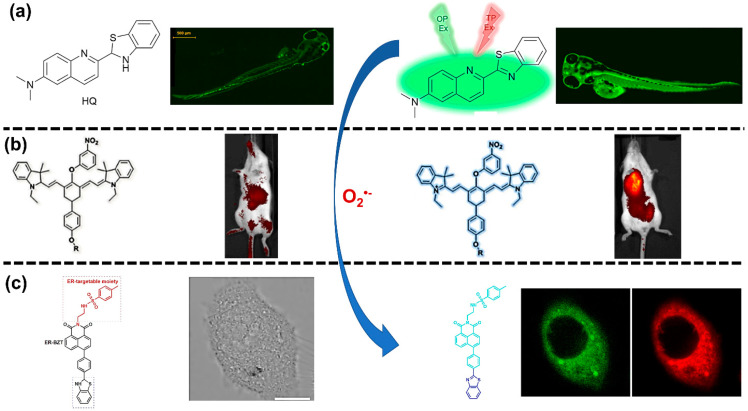
Fluorescent probes before and after interacting with superoxide radical ($O_{2}^{•-})$. (**a**) Two-photon fluorescence images of 6-(dimethylamino)quinoline-2-benzothiazoline in zebrafish (Reprinted with permission from [61] Copyright (2017) Elsevier Publishing). (**b**) Near-infrared fluorescence imaging in murine sarcoma S180 tumor-bearing mice (Reprinted with permission from [62] Copyright (2016) ACS Publications). (**c**) Fluorescence image with two-photon fluorescent probe in human liver cancer (HepG2) cells (Reprinted with permission from [63] Copyright (2017) Elsevier Publishing).

While there are several techniques for the detection of the superoxide radical ($O_{2}^{•-})$, the fluorescence method is one of the most popular for visualizing and tracking the $O_{2}^{•-}$ concentration in live cells because of its high sensitivity, high selectivity, and ease of use. The implementation of fluorescence detection is based on the chemical reaction of $O_{2}^{•-}$ and $O_{2}^{•-}$ released from live cells using either an oxidant-sensitive probe or a non-redox probe [57]. Their interaction will enable the probe to emit fluorescent light, which can be monitored using certain instruments or, in some instances, by the naked eye. This fluorescence emission and the consequent change in probe color can then be used to detect and identify the generation sources of $O_{2}^{•-}$ in live cells. Some examples of this in vivo detection are shown in Figure 1. The implementation of fluorescence for $O_{2}^{•-}$ detection also has certain limitations. One of these crucial limitations for in vivo detection is the biological damage due to high-energy light emission, such as photobleaching [58,59,60].

**Figure 2:**
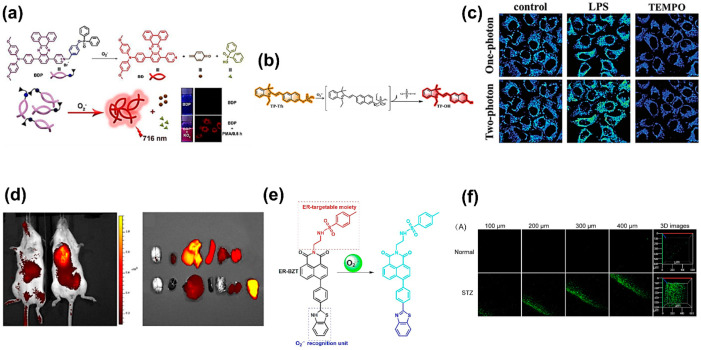
(**a**) Proposed mechanism of superoxide radical $\left(O_{2}^{•-}\right)$ detection by a turn-on near-infrared fluorescent probe (BDP) by Yang et al. (Reprinted with permission from [64] Copyright (2018) Royal Society of Chemistry). (**b**) Mechanism for detection of $O_{2}^{•-}$ with triflates as a fluorescent probe. (**c**) Two-photon imaging in living cells (Reprinted with permission from [68] Copyright (2020) Royal Society of Chemistry). (**d**) In vivo near-infrared (NIR) fluorescence imaging for visualizing $O_{2}^{•-}$ in organs and tumor tissues of mice (Reprinted with permission from [62] Copyright (2016) ACS Publications). (**e**) Endoplasmic-reticulum-targeted two-photon fluorescence (ER-BZT) structure and proposed reaction mechanism for detection of $O_{2}^{•-}$ (**f**) Two-photon fluorescence imaging of $O_{2}^{•-}$ in mouse liver tissues (Reprinted with permission from [63] Copyright (2017) Elsevier Publishing).

Yang et al. [64] designed and developed a near-infrared (NIR) fluorescent sensor by observing the emission at 716 nm and a large Stokes shift at 216 nm, which showed improved signal detection compared to the typical emission wavelength of 450–550 nm. To look at those wavelengths in the presence of $O_{2}^{•-}$, a fluorescent probe was developed based on the luminescence of an aggregation-induced emission (AIE) compound consisting of dibenzo[a,c]phenazine. The initial diphenyl-phosphinyl group of dibenzo[a,c]phenazine in a turn-on near-infrared fluorescence probe (BDP) selectively reacted with $O_{2}^{•-}$ and then ruptured into the pyridinium-modified fluorophore (BD), which became aggregated, emitted NIR light, and exhibited a large Stokes shift, as shown in Figure 2a. Moreover, that sensor showed the ability to detect $O_{2}^{•-}$ in human liver cancer (HepG2) cells, demonstrating the efficacy of the fluorescence method and a suitable wavelength to excite $O_{2}^{•-}$ to fluoresce without causing damage.

As the research for optimizing fluorescent probes continues to advance, there is a prevalent focus on detecting $O_{2}^{•-}$ related to oxidative-stress-induced diseases, such as tumors, diabetes, and liver injury. Although there is a lack of recent publications focused on the connection between $O_{2}^{•-}$ and the development of neurodegenerative diseases, the fluorescence method has been considered a reliable technique for investigating that relationship [65,66]. The interest in cancer research stems from the success in determining the different $O_{2}^{•-}$ concentrations in normal and cancerous tissues by using a fluorescent probe, with cancer cells showing higher levels of $O_{2}^{•-}$ than healthy cells [43,67]. For example, Wang et al. [68] investigated the effect of $O_{2}^{•-}$ on tumor treatment using a two-photon fluorescent probe in combination with triflate as the sensing response unit (Figure 2b). The detection of $O_{2}^{•-}$ was successfully achieved in four minutes without any disturbance from surrounding interfering species, confirming a rapid detection. Furthermore, the use of a two-photon fluorescent probe provided a higher resolution, deeper imaging, and a longer observation time in comparison to a one-photon fluorescent probe, which is shown in Figure 2c. In another study, Huang et al. [62] employed a multi-response fluorescent probe to investigate the relationship between the accumulation of $O_{2}^{•-}$ in biological systems and mitochondrial oxidative stress and cell apoptosis. This novel work demonstrated the ability to locate the presence of $O_{2}^{•-}$ with high fluorescent contrast inside mouse bodies, reaching different depths of tissues and organs, as shown in Figure 2d. Moreover, this fluorescent probe was used in different mouse organs and showed the presence of $O_{2}^{•-}$, specifically in liver and tumor tissues. To do so, the researchers developed two NIR fluorescent probes, Hcy-Mito and Hcy-Biot, for detecting $O_{2}^{•-}$ in mitochondria and tumor tissue, respectively. Those two probes exhibited the same absorption and fluorescence spectra in the presence of $O_{2}^{•-}$ at 770 and 780 nm for absorption and emission, respectively. Both absorption and emission wavelengths were in the NIR region, which is useful for bioimaging of $O_{2}^{•-}$ due to its ability to reduce the interference from cell autofluorescence, lessen photodamage, and profoundly penetrate tissues. Therefore, the studies mentioned here suggest that the fluorescence method has a high potential for distinguishing healthy tissue from cancerous tissue based on the $O_{2}^{•-}$ concentration.

When using the fluorescence method, there are a variety of challenges that need to be overcome, including photobleaching, uneven probe loading [60,69], uncontrollable localization, invasive effects [70], and damage during in situ detection [71]. These problems can be readily subsided by using multi-response fluorescent probes [72,73,74]. The advantages of multi-response fluorescent probes were further highlighted by Gao et al. [75], who studied the influence of $O_{2}^{•-}$ on the tissue inflammatory response under continuous hypoxic conditions. In their work, a fluorescent probe based on a nitrobenzene derivative (HCy-ONO) was developed. The detection of $O_{2}^{•-}$ by the HCy-ONO probe occurred via a hydrogen abstraction reaction to release a relatively low fluorescence emission at 785 nm. The HCy-ONO probe was used to detect $O_{2}^{•-}$ under simulated physiological conditions with a limit of detection (LOD) of 90 nM. Furthermore, their work was able to overcome the aforementioned issues related to spectral overlap interference that occurs during detection. As discussed before, the generation of $O_{2}^{•-}$ within mitochondria has led to studies that investigated the mitochondria’s involvement as a detection site for measuring $O_{2}^{•-}$. For example, Wang et al. [76] successfully targeted $O_{2}^{•-}$ generated in mitochondria in living cells and observed it in both MCF-7 cells (breast cancer cells) and Raw 264.7 cells (tumor cells induced by leukemia) by using a novel mitochondria-targeting probe (DPP-S). Diketopyrrolopyrrole derivatives were employed as sensing molecules in the probe. In the presence of intercellular endogenous $O_{2}^{•-}$ both in vitro and in vivo, the DPP-S emitted fluorescent light at 490 nm, which proved the potential of this type of probe to selectively detect $O_{2}^{•-}$ in both MCF-7 and Raw 264.7 cells. These are only a few examples of the possibilities of $O_{2}^{•-}$ detection using fluorescent probes to successfully detect and monitor cancer and tumor cells within the mammalian body, as commonly demonstrated in mice.

Furthering the scientific understanding of the relationship between the concentration of $O_{2}^{•-}$ and diabetes relies on the development of effective in vivo detection techniques, and fluorescence imaging arises as a promising method to study that relationship. It has been found that protein accumulation in the endoplasmic reticulum (ER) leads to oxidative stress conditions that result in the development of metabolic diseases, such as diabetes [77,78]. By targeting the ER as the original source of metabolic diseases, two-photon fluorescent probes were successfully used to image the fluctuation of $O_{2}^{•-}$ and to further elucidate the connection between $O_{2}^{•-}$ and ER-associated disorders. The fluorescent probe designed and fabricated by Xiao et al. [63] used benzothiazoline as the selective sensing element, which was conjugated with methyl sulphonamide as the ER-targetable moiety. The probe emitted strong fluorescence in the presence of $O_{2}^{•-}$ at 450 nm compared to 375 nm in the system containing no $O_{2}^{•-}$. This is due to the dehydrogenation reaction of benzothiazoline with $O_{2}^{•-}$. The detection mechanism of $O_{2}^{•-}$ using this novel fluorescent probe is shown in Figure 2e. Xiao et al. compared normal mice, diabetic mice, and drug-treated diabetic mice to prove the possibility of using their developed fluorescent probe to detect the presence of $O_{2}^{•-}$ in liver tissue. As shown in Figure 2f, the 3D stack demonstrated that the liver tissue of diabetic mice induced by streptozotocin (STZ) had a higher fluorescent intensity than normal mice (normal), which could be attributed to higher concentrations of $O_{2}^{•-}$. Therefore, this type of two-photon fluorescent probe shows promising ability for monitoring the presence of $O_{2}^{•-}$ in live cells.

**Figure 3:**
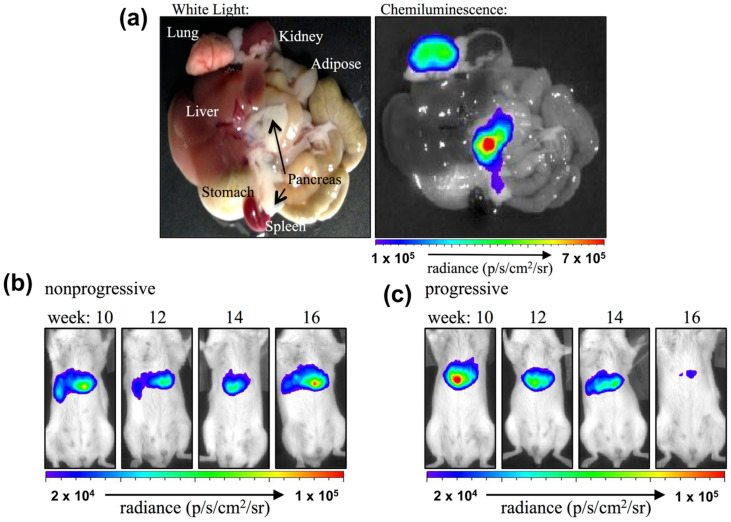
(**a**) Ex vivo imaging of chemiluminescence resulting from the pancreas and lungs and images of a representative (**b**) nonprogressive mouse and (**c**) progressive mouse at 10, 12, 14, and 16 weeks of age (Reprinted with permission from [79] Copyright (2016) Public Library of Science).

It is known that post-prandial hyperglycemia can occur in both healthy and insulin-resistant individuals. With this understanding, Bronsart et al. [79] pioneered the detection of $O_{2}^{•-}$ using a chemiluminescent probe to determine the effects of transient post-prandial hyperglycemia and evaluate whether the concentration of $O_{2}^{•-}$ is physiologically involved. To do so, they measured the chemiluminescence from the interaction between $O_{2}^{•-}$ in beta cells and coelenterazine (sensing enzyme) in mice with and without hyperglycemia. Their probe effectively detected the presence of $O_{2}^{•-}$ in live pancreas and lung tissues with fluorescence emission at 520 nm, as shown in Figure 3a. Moreover, as demonstrated in Figure 3b,c, the chemiluminescent probe was able to differentiate mice with (progressive group) and without hyperglycemia (nonprogressive group) based on $O_{2}^{•-}$ concentrations. When comparing both groups over certain times, Bronsart et al.’s chemiluminescent probe revealed that the nonprogressive group did not show any fluctuation of the $O_{2}^{•-}$ concentration in beta cells (Figure 3b). Nonetheless, the progressive group showed slow attenuation of the chemiluminescence signal over time, from the beginning of hyperglycemia development until the 16th week (Figure 3c). This experimental result significantly proved the hypothesis that physiological hyperglycemia is caused by continuous exposure to low concentrations of $O_{2}^{•-}$ in cells. Additionally, this study successfully identified $O_{2}^{•-}$ as a biomarker for the beta cell mass in pancreas, predicting its susceptibility for diabetes [79]. Therefore, this work provided a direct validation of the chemiluminescence method to monitor dynamic changes in the $O_{2}^{•-}$ concentration in cells and organisms.

Another area of interest in $O_{2}^{•-}$ research is related to drug-induced acute hepatotoxicity of the liver. Although fluorescence imaging can be used in biological systems for the detection of $O_{2}^{•-}$, it is still necessary to increase the sensitivity and selectivity of fluorescent probes to track signal changes within a liver injury. The ER is an important membrane-bound organelle that has been proven to be involved in drug-induced acute liver injuries [80,81]. When observing the relationship between ER stress, increased $O_{2}^{•-}$ levels, and resulting liver injury, fluorescent probes have demonstrated the ability to visualize $O_{2}^{•-}$ changes involved in this type of liver injury, and serve as a useful tool for liver disease diagnosis and treatment [82]. For the development of novel fluorescent probes, the efficiency of the fluorescence method in the detection of $O_{2}^{•-}$ in HepG2 cells has been verified. Their use in real-life applications justified the continued development of probes, including the fabrication of NIR fluorescent probes that are able to improve the selectivity and decrease the cytotoxicity and cell damage during $\;O_{2}^{•-}$ detection [64]. However, it is still necessary to better understand the relationship between $O_{2}^{•-}$ species and oxidative stress diseases, and this requires more sensitive and selective fluorescent probes for more in-depth studies.

**Figure 4:**
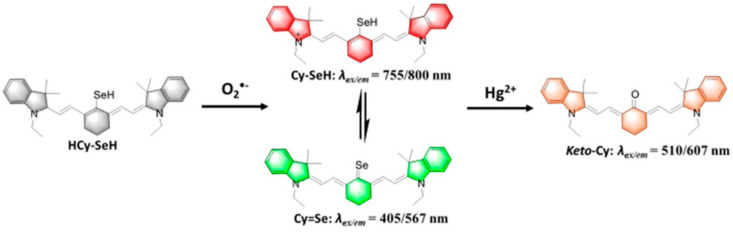
Proposed mechanism for the associated detection of superoxide radical ($O_{2}^{•-}$) and mercury (II) ion (Hg^2+^) (Reprinted with permission from [83] Copyright (2018) ACS Publications).

Although $O_{2}^{•-}$ is considered the primary ROS due to its abundance and involvement in the production of other ROS, this is not its only role. Actually, $O_{2}^{•-}$ is involved in physiological processes beyond the generation of other ROS, and novel discoveries have been made around the connection between $O_{2}^{•-}$ and various biological processes. For example, Wang et al. [83] developed an NIR three-channel fluorescent probe for the associated detection of $O_{2}^{•-}$ and mercury (II) ions (Hg^2+^) in cells and mouse models of chronic mercury poisoning. The proposed mechanism, as shown in Figure 4, was based on two chemical reactions of a heptamethine cyanine fluorophore modified with a selenol (-SeH) group. In the presence of $O_{2}^{•-}$, the NIR cyanine-fluorophore-modified probe released light emission at 740 and 420 nm. This light emission was caused by the reduction reaction of the heptamethin cyanine fluorophore in the fluorescence probe via a common reaction of hydrogen abstraction. Their developed NIR cyanine fluorophore probe provided additional advantages in terms of improved tissue penetration and reduced interference species. Remarkably, the fluorescent probe could be used in live cells and provided solid evidence of the accumulation of Hg^2+^ that interrupted the function of cellular antioxidants, causing the overproduction of $O_{2}^{•-}$ in cells. This work is an excellent example of the effectiveness of fluorescence methods for the detection of $O_{2}^{•-}$ in live cells; however, the feasibility of this technique in a real physical environment is questioned. As believed by several researchers, fluorescent-dependent techniques are considered a one-time detection procedure; consequently, these techniques can misrepresent the total $O_{2}^{•-}$ concentration generated in living cells [79,84].

### 2.3. Electrochemical Method for Superoxide Radical ${(O}_{2}^{•-})$ Detection

Some drawbacks of the fluorescence method are that it is expensive [85], time-consuming, nonportable [86], requires a large space [87], possesses limited temporal or spatial resolution [88], and is a single-use detection method. To overcome these challenges, researchers have investigated the possibility of using an electrochemical technique for the detection of the superoxide radical ($O_{2}^{•-}$), which has proven effective in monitoring unstable $O_{2}^{•-}$ concentrations at the generation source. Electrochemical techniques employ the same principle as the fluorescence method as they combine electrochemical reactions with specific sensing elements to selectively detect $O_{2}^{•-}$. Several publications between 2016 to 2020 showed the detection of $O_{2}^{•-}$ in cancerous cells [89,90], tumorous cells [91,92], macrophages [93,94], skeletal muscle cells, human glioblastoma cells, and human keratinocyte cells [47]. However, there are still several limitations that need to be considered when implementing electrochemical techniques for $O_{2}^{•-}$ detection, especially in tissues. Cell penetration is one of the requirements to be addressed during the development of electrochemical methods for $O_{2}^{•-}$ detection. Since $O_{2}^{•-}$ is generated in mitochondria and has high reactivity with a short lifetime [95], the design of electrochemical method must be both biologically and chemically compatible to allow cell penetration and, ultimately, $O_{2}^{•-}$ detection at its original source. Moreover, sensitivity and selectivity are important parameters as the concentration of generated $O_{2}^{•-}$ in cells and tissues is extremely low (picomolar to nanomolar range) [95]. Lastly, biocompatibility is also of great importance since cells and tissues are vulnerable to nonbiological materials, which could lead to cell death [96,97].

To provide electrochemical techniques with high selectivity, sensitivity, and biocompatibility, biological catalysts (enzymes), such as superoxide dismutase (SOD) and cytochrome-c (Cyt-c), are commonly used in electrochemical methods for the detection of $O_{2}^{•-}$ in cells and tissues. These special enzymes, which act as a cellular antioxidant defense system in the human body, allow fast dismutation of $O_{2}^{•-}$ [98]. To improve the sensitivity of electrochemical techniques, biological molecules are commonly used together with metal oxide nanomaterials. Since most metal oxide nanoparticles have high conductivities and high surface areas for biological molecules to attach on, the number of reactive sites from biological molecules and the electrical communication between the electrode and the redox centers are significantly improved [99,100].

**Figure 5:**
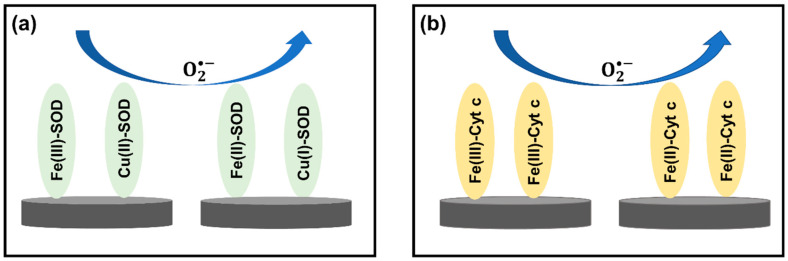
Redox reactions between (**a**) superoxide dismutase (SOD) and superoxide radical ($O_{2}^{•-}$) (Adapted with permission from [105] Copyright (2002) Elsevier Publishing) and between (**b**) cytochrome-c (Cyt-c) and $O_{2}^{•-}$(Adapted with permission from [106] Copyright (1999) John Wiley and Sons).

Additionally, the combination of nanomaterials with biological molecules needs to be carefully chosen to ensure its biocompatibility for biomedical applications. As the properties of electrochemical sensors depend on sensing elements on the electrode surface, materials such as hydrogel [101] and chitosan [102] are frequently deposited on the surface to enhance biocompatibility. In addition, these biocompatible materials also preserve the inherent catalytic properties of biological molecules [101]. Even though the biological molecules are combined with nanomaterials to improve the sensitivity, the principal redox reactions between the biological molecules and $O_{2}^{•-}$ remain unchanged, as shown in Figure 5. SOD is a well-known metalloenzyme and requires bonding with a metal cofactor to initiate the SOD mechanism. Iron (Fe), zinc (Zn), copper (Cu), and manganese (Mn) are common metal ions found together with SOD [103]. A proposed mechanism between SOD and $O_{2}^{•-}$ is demonstrated in Figure 5a. With regard to Cyt-c, this metalloprotein contains an iron-centered porphyrin in its structure [104]. The Fe ions easily react with $\;O_{2}^{•-}$ through the oxidation reaction shown in Figure 5b. Therefore, the redox reaction between biological molecules and $O_{2}^{•-}$ can be used to determine the concentration of $O_{2}^{•-}$ in the biological systems of interest.

The main disadvantage of electrochemical enzyme-based sensors, however, is that the operation at a high potential induces interference signals from other coexisting electroactive species. As such, biological enzymes on the electrode surface have been frequently modified with either organic or inorganic materials to reduce the operating potential. For example, Crulhas et al. [89] strengthened the performance of their electrochemical sensor to investigate details of the cellular dynamics within oxidative-stress-induced diseases. To do so, they modified the surface of a microelectrode with a polyethylene glycol diacrylate hydrogel matrix containing SOD and ferrocene (Fc) as a sensing element. This polymer matrix/enzyme electrochemical biosensor provided a reliable electrocatalytic response with high sensitivity and selectivity, which was attributed to the specific reaction between SOD and $O_{2}^{•-}$. Fc in the hydrogel matrix also facilitated the redox-stable states in the electrochemical biosensor. Consequently, the developed electrochemical biosensor successfully detected $O_{2}^{•-}$ released from prostate cancer cells in vitro and in real time in the range of 3 nM to 4.17 µM [89]. This detection method is regarded as a promising technology for monitoring $O_{2}^{•-}$ released from living cells.

**Figure 6:**
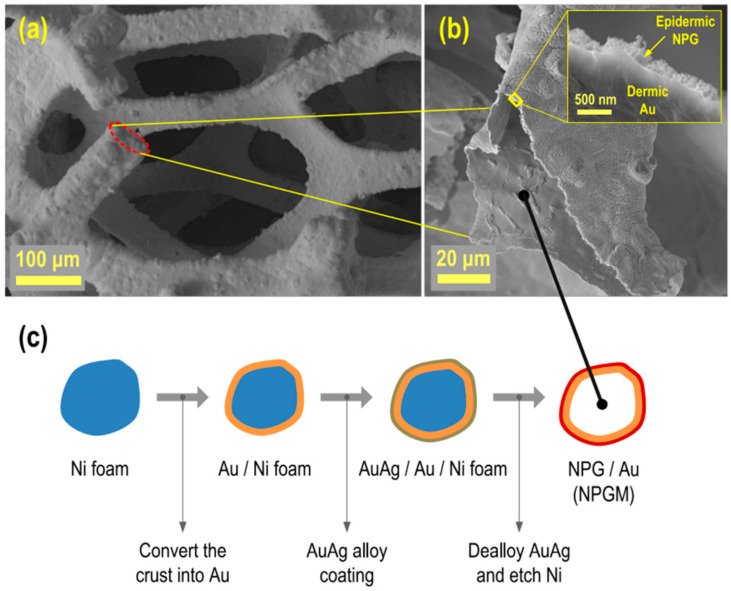
(**a**,**b**) Scanning electron microscopy (SEM) images showing the bilayer structure of tubular ligaments and (**c**) schematic view of the cross section of a ligament, showing the fabrication steps leading to the final nanoporous gold mesh (NPGM) structure (Reprinted with permission from [107] Copyright (2017) Elsevier Publishing).

The main challenge associated with live/online monitoring is the minuscule concentration of $O_{2}^{•-}$ released from living cells, such as skeletal muscle tissues, in the orders of pico- to nanomolar concentrations. To succeed in detecting low concentrations of $O_{2}^{•-}$ via an electrochemical method, Sadeghian et al. [107] developed an innovative working electrode with a 3D macroporous mesh of nanoporous gold, as shown in Figure 6c. Figure 6a,b shows the success of the synthesis protocol. One of the advantages of this electrode is the tremendous increase of the surface area for dispersion of the sensing element (Cyt-c) that facilitates the interaction with $O_{2}^{•-}$, thus improving the limit of detection (LOD) to picomolar levels.

Most of the development has been directed toward improving the enzyme-based sensors and their pitfalls by using inorganic materials, as incorporation of highly conductive materials into an electrochemical sensor has proven to increase the sensor’s ability to detect $O_{2}^{•-}$ at the nanomolar range. Although enzyme-based sensors can perform stable detection of $O_{2}^{•-}$, the enzyme itself still faces a couple of challenges, such as the deactivation and inconsistent reproducibility of the sensor performance under harsh conditions. Moreover, $O_{2}^{•-}$ is typically released from living cells in the nanomolar range and rapidly diffuses away from its generation source; thus, electrochemical biosensors need to be placed in close proximity for the detection of $O_{2}^{•-}$. This requirement adds another challenge for in vivo detection, as the sensor introduces a foreign inorganic or metal component with poor biocompatibility that can lead to an immune response and further inflammation [108,109]. In this regard, biocompatible materials have also been explored alongside sensing molecules on the electrode surface.

While enzyme-based sensors have great selectivity and sensitivity, these advantages are overshadowed by the high cost, short lifetime, poor reproducibility, and vulnerability of the enzymes in harsh operating environments [47]. Poor long-term stability is one of the most important and unavoidable issues because natural enzymes are prone to denaturation under different external conditions, such as pH, temperature, and humidity [110]. To address these challenges, there have been a multitude of emerging technologies, such as enzyme-free electrochemical sensors, which show great potential for stable detection under harsh conditions. These enzyme-free electrochemical sensors use a variety of materials (nanotube structures, nanocomposites, and catalysts, among others) and nanocomposite preparation methods [111]. Synthetic biomimetic enzymes have also proven to be good alternatives, typically acting as low-cost artificial enzymes displaying improved stability and excellent recyclability [47,110,112].

Some challenges associated with the construction of electrochemical sensors for in situ $O_{2}^{•-}$ detection include the inability to maintain the desired catalytic activity and low electrical conductivity, both of which are critical for good sensor performance. In 2016, Liu et al. [113] developed an enzyme- and metal-free electrochemical sensor for the detection of $O_{2}^{•-}$ via interaction with the surface of nitrogen-doped hollow mesoporous carbon spheres (N-HMCS). Nitrogen doping proved to be the main contributing factor for high sensitivity due to the strong electron donor nature of nitrogen that contributed to the enhanced electron transport properties and chemical reactivities. However, the reaction mechanism of $O_{2}^{•-}$ on the surface of N-HMCS was not elucidated. Other parameters, such as good conductivity, large pore size/volume, and large specific surface area, also granted the sensor with improved sensitivity toward $O_{2}^{•-}$ with a LOD of 2.2 µM. Unfortunately, the LOD with this metal- and enzyme-free electrochemical sensor is not low enough to detect the presence of $O_{2}^{•-}$ in live cells, which is in the nanomolar range. As a result, several metal-oxide-based electrochemical sensors have been developed to further improve both sensor sensitivity and sensor selectivity.

**Figure 7:**
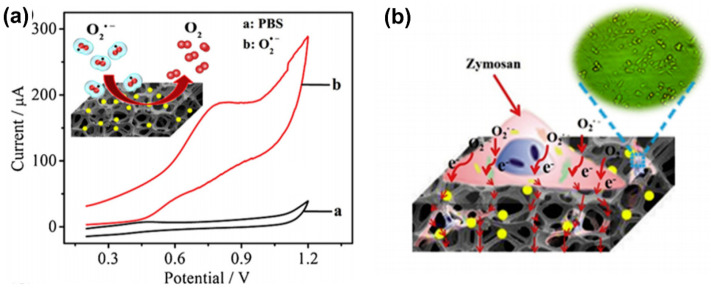
(**a**) Cyclic voltammetry response of platinum nanoparticles on a 3D graphene foam (Pt@GF)/glassy carbon electrode (GCE) in phosphate-buffered saline (PBS) with (curve b) and without (curve a) 39 μM of superoxide radical ($O_{2}^{•-}$) adsorption and oxidation processes and (**b**) scheme showing the zymosan (Zym)-triggered $O_{2}^{•-}$ production in a cell (Reprinted with permission from [114] Copyright (2017) Elsevier Publishing).

Hu et al. [114] developed an electrochemical sensor using platinum nanoparticles (PtNPs) deposited onto 3D graphene foam (GF) as an electrode material. The 3D GF support was selected because the metal nanoparticles can retain their positive charge to interact with $O_{2}^{•-}$ resulting in improved sensor sensitivity. The 3D GF was also reported to be a biocompatible platform with a high surface area and suitable cell adhesion/growth for in situ $O_{2}^{•-}$ detection. Additionally, it was found that the deposition of PtNPs on 3D GF decreased the repulsion between the negatively charged $O_{2}^{•-}$ and the electrode surface, leading to a higher electron transfer rate and better electrocatalytic activity than bulk 3D GF. The combination of PtNPs and 3D GF also provided a well-defined and uniformly shaped oxidation current toward $O_{2}^{•-}$ in cyclic voltammetry (CV), as shown in Figure 7a, and the synergy between them improved the sensitivity of the sensor by 140%, with a lower LOD of 10 nM and a rapid sensor response of 3.6 s. Remarkably, when applied to zymosan (Zym)-triggered $\;O_{2}^{•-}$ in human melanoma cells, as shown in Figure 7b, the sensor was able to monitor 680.03 nM of $O_{2}^{•-}$ released from cells in situ. This work demonstrated the potential of integrating metal oxide nanoparticles with highly conductive materials for the detection of $O_{2}^{•-}$ with high sensitivity and selectivity [114].

**Figure 8:**
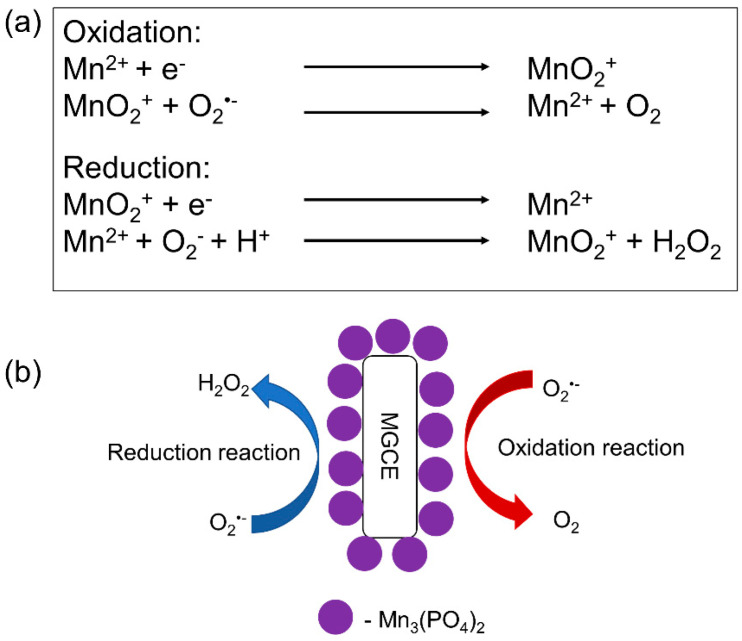
(**a**) Oxidation–reduction reaction between superoxide radical ($O_{2}^{•-}$) and Mn_3_(PO_4_)_2_ and (**b**) schematic of the redox reaction occurring on the surface of the modified magnetic glassy carbon electrode (MGCE) (Reprinted with permission from [110] Copyright (2017) Elsevier Publishing).

Another option is to use synthetic biomimetic enzymes as a sensing element. As previously stated, synthetic biomimetic enzymes provide various advantages over natural enzymes, such as a long-term stability and resistance to severe environments. Among the investigations of synthetic biomimetic enzymes and their ability to improve the performance of electrochemical sensors for the detection of $O_{2}^{•-}$, one of the most interesting ones is that by Peng et al. [110], who immobilized Mn_3_(PO_4_)_2_ nanoparticles on the surface of magnetic polystyrene nanotubes to modify the surface of a magnetic glassy carbon electrode (MGCE). Mn_3_(PO_4_)_2_ nanoparticles were used as a synthetic biomimetic enzyme (Mn superoxide dismutase (MnSOD)), i.e., the sensing element of the sensor electrode, whereas polystyrene nanotubes acted as electron carriers, while providing Mn_3_(PO_4_)_2_ with a high surface area for their self-assembly. The interaction between $O_{2}^{•-}$ and Mn_3_(PO_4_)_2_ is shown in Figure 8, with $O_{2}^{•-}$ conversion catalyzed by Mn_3_(PO_4_)_2_ on the surface of the developed sensor. They concluded that the use of MGCE tremendously improved the electron transfer rate between the electrode surface and Mn_3_(PO_4_)_2_. The integration of highly selective Mn_3_(PO_4_)_2_ and the highly conductive MGCE enabled the fabrication of this synthetic biomimetic enzyme-based sensor to quantify the concentration of $O_{2}^{•-}$ released from prostate cancer cells, being this of 0.079 μM.

Another example of a biomimetic enzyme-based sensor was proposed recently by Zhao et al. [115], who synthesized a Co-based nanocomposite as an alternative to a biological enzyme for $O_{2}^{•-}$ detection in human umbilical vein endothelial cells (HUVECs). The main reasons for using Co-based nanocomposites with SOD mimetic electrocatalytic activity are to overcome the poor electron transfer between the redox centers of SOD and the electrode surface and to enhance the sensor durability and repeatability. Remarkably, this Co-based nanocomposite electrochemical sensor successfully detected $O_{2}^{•-}$ at a low electric potential of 0.2 V, which significantly reduced the interference signals from other electrochemical active species. Furthermore, the authors demonstrated that their microemulsion method was able to provide the Co-based nanocomposites with large surface areas, porous structures, and unique superarchitectures, which led to overall improved performance with a LOD as low as 1 nM. Additionally, they successfully detected the presence of $O_{2}^{•-}$ in HUVECs in real time. Therefore, it can be concluded that electrochemical techniques have great potential for in situ real-time monitoring of $O_{2}^{•-}$ for clinical diagnosis and drug therapy analysis [116,117,118].

## 3. Hydrogen Peroxide (H_2_O_2_) Detection

### 3.1. Introduction to Hydrogen Peroxide (H_2_O_2_) Detection

As previously mentioned, reactive oxygen species (ROS) are formed under normal physiological conditions and can provide both beneficial and detrimental effects in cellular processes. One of the most interesting ROS is hydrogen peroxide (H_2_O_2_), which is also the most found ROS in industrial and medical setups. The generation of H_2_O_2_ in mitochondria was first discovered in 1966 [119], and since then, several works have investigated its functions and concluded that it is a simple, important, and powerful oxidant required in biological functions involving intercellular signaling and recruitment of immune cells, in addition to helping cells with shape changes, such as cell elongation and differentiation [120,121]. Most ROS are short-lived molecules with half-lives of nanoseconds. However, H_2_O_2_ has a longer lifetime comparatively, reaching up to minutes, depending on the surrounding levels of H_2_O_2_-decomposing enzymes. Therefore, H_2_O_2_ is able to cross cell membranes, diffuse into different cellular compartments, and may even act as a second messenger in the cell signal transduction due to the oxidation of protein thiols, specifically the cysteine thiol in signaling proteins [45,122,123,124]. However, to take advantage of H_2_O_2_ in biological functions, its concentration needs to be below 100 nM, as levels above this lead to oxidative stress and damage [120] that may cause severe diseases and related conditions, such as cancer, diabetes, Alzheimer’s and Parkinson’s diseases, and even accelerated aging [125].

Although H_2_O_2_ reacts poorly with most biological molecules due to its kinetically driven reactions, its detrimental effects stem from the highly reactive •OH that are generated from exposure to ultraviolet light in the presence of transition metals, such as ferrous ions via the Fenton reaction [126]. Therefore, H_2_O_2_ itself is not the direct cause of these severe diseases, if not for the production of •OH from H_2_O_2_ via the Fenton reaction in cells. Due to its accessibility, high availability, and major role in disease processes, H_2_O_2_ is a widely used chemical for studying oxidative stress, and it has become an integral part of the current knowledge of this field of study. While H_2_O_2_ has shown to be beneficial in cell regeneration, there is also a direct link between abnormal H_2_O_2_ production and the occurrence of diseases [127]. Thus, H_2_O_2_ could be used as a biological marker to indicate the development of various oxidative-stress-related diseases. H_2_O_2_ is also one of the most extensively researched ROS, and its detection has mostly taken place using methods such as spectrophotometry, fluorescence, chemiluminescence, and electrochemistry [128]. Interestingly, within the past five years, electrochemical techniques for the detection of H_2_O_2_ have gained increased attention due to their simple and easy operation, good sensitivity, and high selectivity with a short analytical time. For that reason, this section will mainly focus on the electrochemical technology.

### 3.2. Electrochemical Techniques for Hydrogen Peroxide (H_2_O_2_) Detection

For the detection of hydrogen peroxide (H_2_O_2_) by electrochemical techniques, the electrode surface needs to be modified with an electrocatalyst that can be either an inorganic or an organic biological compound, such as cytochrome-c (Cyt-c), horseradish peroxidase (HPR), hemin, or myoglobin. As for biological molecules, they contain the redox agent heme, which acts as an active catalytic element for H_2_O_2_ detection. Then, an electrochemical method measures an increased redox response (in the form of current or impedance change) due to the direct electron transfer (DET) between the active sites reacting with H_2_O_2_ and the electrode surface [129]. However, the DET between the heme-containing biological molecules and the electrode surface is slow, which is likely due to the position of the redox-active centers deep inside the molecule structure and the protein shell on biological molecules [130,131]. The oxidative self-degradation and the aggregation of biological molecules could also affect the efficiency of electrochemical techniques for H_2_O_2_ detection.

An alternative approach to overcoming these challenges is immobilization of heme-containing biological molecules on highly conductive materials with a large surface area. For example, different combinations of metal/metal oxide nanoparticles (NPs) and biological elements have been reported in the literature for improved detection of H_2_O_2_ [101,132,133]. These metal and metal oxide NPs are well known for their high conductivities, ability to provide a large surface area, and good compatibility with biological molecules for their immobilization without compromising the peroxidase properties. Additionally, these NPs help to prevent the aggregation of biological molecules. Among the different materials, gold nanoparticles (AuNPs) have been frequently integrated with biological molecules to increase the enzyme loading, promote suitable enzyme orientations, and decrease the distance for the electron transfer between the electroactive centers of the organic molecules and the underlying electrode. For example, Zhang et al. [134] combined Cyt-c with AuNPs to take advantage of the large specific surface area, excellent electron conductivity, and good biocompatibility. Their sensor showed improved sensitivity with a limit of detection (LOD) as low as 0.3 µM. They concluded that AuNPs can retain the native structure of Cyt-c and enhance the electrocatalytic characteristics of Cyt-c on the electrode surface for H_2_O_2_ detection [134].

Several other conductive materials have been combined with biological electrocatalysts to improve the DET for enhanced sensitivity and selectivity. For example, Aghamiri et al. [135] immobilized Cyt-c on an electrode modified with a polymer-based nanocomposite. It was hypothesized that the high electrical conductivity of the polymer-based nanocomposite could improve the DET between the redox-active centers of the sensing protein and the electrode surface. Therefore, the researchers proposed a bilayer of a conducting polymer film of polyaniline/polypyrrole (PAN/PPY) as an interlayer between Cyt-c and the electrode surface to facilitate the DET. To improve the mechanical strength of the polymer film, the conductive bilayer polymer was deposited inside carboxylated multi-walled carbon nanotubes (cMWCNTs), and the Cyt-c/PAN/PPY/cMWCNTs material showed an improved DET with a LOD of 0.1 µM.

**Figure 9:**
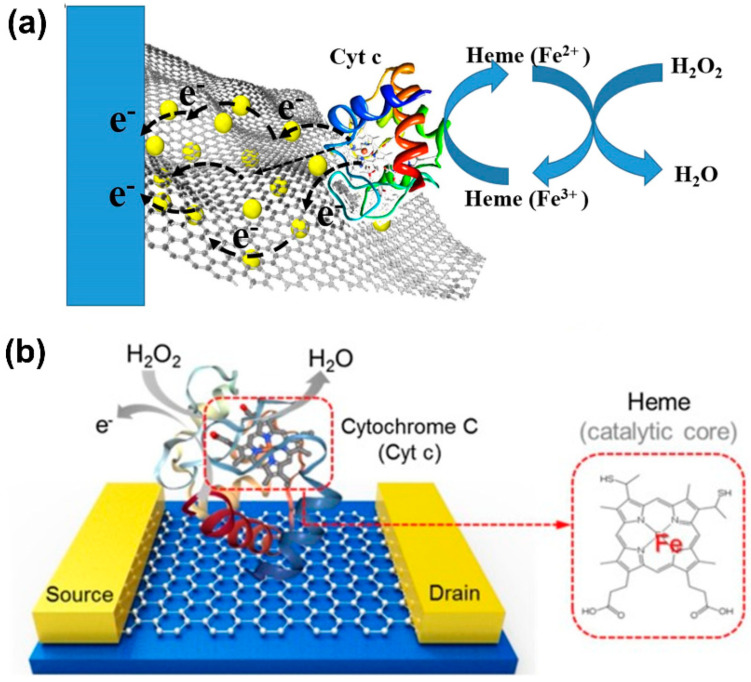
(**a**) 3D graphene aerogel decorated with Au nanoparticles with immobilized cytochrome c on a glassy carbon electrode (AuNPs/Cyt-c/GCE) sensor for the detection of hydrogen peroxide (H_2_O_2_) (Reprinted with permission from [145] Copyright (2019) Elsevier Publishing). (**b**) Cyt-c-decorated graphene field-effect transistor for H_2_O_2_ detection (Reprinted with permission from [146] Copyright (2020) Elsevier Publishing).

Other materials, such as graphene oxide [136,137,138], carbon nanotubes [139,140], ZrO_2_ [141], Ni foam [142], ZnO [143], and TiO_2_ [144], have also provided promising results. For example, as shown in Figure 9a, Zhao et al. [145] integrated Cyt-c with AuNPs on 3D graphene aerogel to remedy the effect of poor DET, and Lee et al. [146] employed a combination of graphene oxide as a conductive material with Cyt-c for the detection of H_2_O_2_ (Figure 9b).

As previously mentioned, other heme proteins such as hemin (HN), myoglobin, and hemoglobin (Hb) also have potential as active biological catalytic species for H_2_O_2_ detection [147,148,149]. Myoglobin and hemin are usually immobilized on AuNPs due to their good conductivity, excellent biocompatibility, and the ability to easily interact with proteins via the thiol groups and disulfide bonds, leading to a simple biosensor preparation. For example, in 2016, Xu et al. [150] took advantage of AuNPs to improve the sensor efficiency for the detection of H_2_O_2_ using Hb as the biological redox protein. To do so, they immobilized Hb on a magnetic Fe_3_O_4_/AuNPs nanocomposite. Their objective was to improve the DET, while maintaining the biological activity of Hb on the electrode surface. The immobilization of Hb on Fe_3_O_4_/AuNPs resulted in a significant improvement of the overall sensor performance due to the enhanced DET and higher exposure of redox-active sites to H_2_O_2_, with a LOD of 0.1 µM.

As previously stated, graphene oxide (GO) and carbon nanotubes (CNTs) have also been frequently combined with biological redox proteins. For example, hemin was immobilized on either GO or CNTs to reduce the challenges associated with its use for the detection of H_2_O_2_ [151,152,153]. For instance, Le et al. [153] combined both thermally reduced GO (TRGO) and CNTs with hemin (hemin-TRGO-CNTs) to modify an electrode for H_2_O_2_ detection. While TRGO can provide biomolecules recognition, CNTs provide proteins with a large aspect ratio and good conductivity for electrolyte ions to diffuse into the electrochemical device. The combination of TRGO and CNTs could form a firm π–π interaction with redox biological proteins, and this synergy could also provide increased solubility and dispersibility, and reduced aggregation, resulting in a greater surface area between the redox biological proteins and the electrode substrate. This hemin-TRGO-CNT-modified electrode was successfully used for detection of H_2_O_2_ with a LOD of 0.0933 mM [153]. Another example was provided by Kong et al., who improved sensor sensitivity by loading hemin on a 3D building block of GO and single-walled carbon nanotubes (SWCNTs). Remarkably, the great synergy between hemin, GO, and SWCNTs highly improved the sensor performance, with a LOD as low as 0.05 μM [151], which was lower than that obtained with either GO or SWCNTs [154,155,156,157]. On a related note, Deac et al. [158] demonstrated that hemin was polymerized on a GO-modified electrode, which is believed to improve both the catalytic activity and electrochemical stability. Interestingly, the sensitivity of the hemin-polymerized electrode increased up to 61.94 ± 6.35 mA·M^−1^, which was higher than that obtained with directly deposited redox proteins [159,160,161].

Porous materials have also been used for immobilization of redox proteins. Various conductive complex materials, such as Ag-Au [162], Fe_3_O_4_-Au [150], polyethylenimine (PEI)-Au [163], and GO-chitosan-Au [159,160,161,164], have been reported in the literature. As previously mentioned, one of the main reasons for integrating redox proteins with conductive substrates with large surface areas is to improve the amount and stability of bound redox proteins on the electrode surface. For example, Zhao et al. [165] developed Fe-hemin–metal organic frameworks (MOFs) supported on a chitosan-reduced GO glassy carbon electrode (Fe-hemin-MOFs/CS-rGO@GCE) for the detection of H_2_O_2_ in live cells. The Fe-MOF complex was employed as a support to firmly activate hemin, while CS-rGO was used to amplify the electrochemical signal. Due to the advantages of both Fe-MOFs and CS-rGO, the hemin-based electrochemical sensor was able to detect H_2_O_2_ released from a human serum sample and MCF-7 cells (breast cancer cells) in real-time monitoring, with an estimate of 1.1 μM of H_2_O_2_ released from each living cell. However, one of the main concerns with Fe-MOFs as a base substrate is the potential generation of harmful free radicals, e.g., •OH, from the interaction between Fe^2+^ and released H_2_O_2_ (Fenton reaction), which could lead to the deformation of the Fe-MOF structure, disrupting the sensor operation.

An alternative method for the development of redox-protein-based electrochemical sensors for live cells was proposed by Wang et al. [166], who modified an electrochemical sensor with a composite of hemin, AuNPs, and reduced GO. In this case, they also used chitosan due to its outstanding permeability and high adhesiveness, which could form a stable film on top of the electrode surface. To do that, chitosan was mixed with the composite to fasten the redox proteins on the electrode surface. The use of hemin combined with highly biocompatible AuNPs, conductive reduced GO, and permeable chitosan made it possible to detect H_2_O_2_ with a LOD of 9.3 nM.

Even though the combinations of redox proteins and conductive materials have proven to be effective for the detection of H_2_O_2_, further in-depth studies are still needed to prevent the degradation of redox proteins under harsh environmental conditions. The difficulties associated with the use of biological sensing molecules have motivated researchers to use inorganic materials with mimicking enzymatic properties for H_2_O_2_ detection. Some of those materials are silver NPs (AgNPs) [167,168,169,170], AuNPs [171,172,173,174], platinum NPs (PtNPs) [175,176], ZnO and ZnMn_2_O_4_ [177,178], Ag and Au on ZnO nanoflowers [179], MnO_2_ and Mn_3_O_4_ [180,181], Co_3_O_4_ [182,183], Co_9_S_8_ hollow spheres [184], Cu_3_P nanowires [185], and lanthanide coordination polymer NPs [186].

Unfortunately, even though these inorganic materials possess several advantages over enzyme-based electrochemical sensors, some of them also suffer from aggregation of NPs, resulting in decreased sensor sensitivity [143,179]. For that reason, several investigations have focused on developing synthesis methods to prevent the aggregation on electrode surfaces. Among them, AgNPs have been widely used due to their reasonable cost, large surface area, and excellent activity and stability. Different techniques have been employed to disperse AgNPs on electrode surfaces to avoid their aggregation. For example, Goud et al. [187] deposited AgNPs on a scaffold layer of polyethylene glycol (PEG) and hexamethylenediamine (HMDA), which was immobilized on a screen-printed carbon electrode (SPCE). The main function of the scaffold layer was to control the distribution of AgNPs on the electrode surface, which resulted in lower aggregation, generation of large tunnels that can accommodate the electron transfer during the electrodeposition of AgNPs, and increased availability of active sites for the reduction of H_2_O_2_. This sensor was used to analyze the content of H_2_O_2_ in a toothpaste product, with a LOD of 1.5 µM.

In addition to the dispersion of NPs, morphology is another critical parameter that needs to be considered in the development of electrochemical sensors for H_2_O_2_ detection. For example, Preethi et al. [188] studied the effect of the morphology of AgNPs on the sensor sensitivity toward H_2_O_2_ detection. To do so, they used chitosan to control the nucleation and growth process of Ag deposition on the transducer surface. In their investigation, AgNPs deposited with chitosan as a structure-directing agent had well-defined morphology, improving the sensor sensitivity toward H_2_O_2_ by up to 0.06 µM. This work highlighted the importance of both the morphology and the distribution of metal NPs for sensor efficiency.

**Figure 10:**
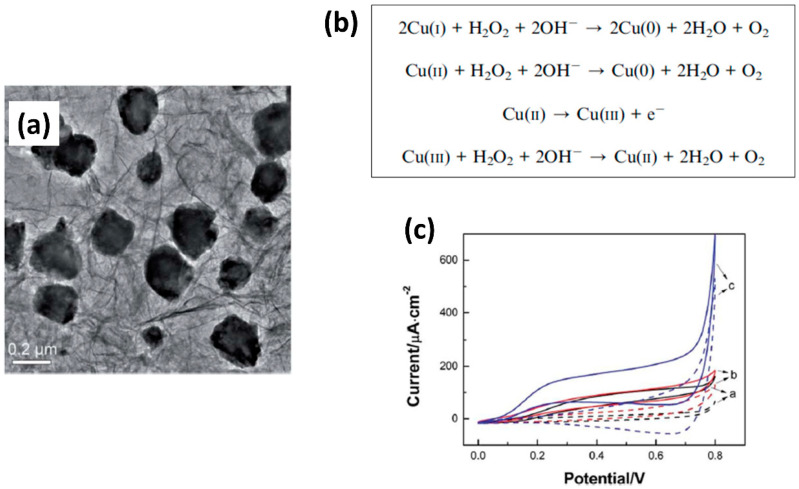
(**a**) Transmission electron microscopy (TEM) image of Cu_2_O/CuO–reduced graphene oxide (Cu_2_O/CuO@rGO) composite after thermal treatment at 600 °C, (**b**) oxidation mechanism of Cu_2_O and CuO with hydrogen peroxide (H_2_O_2_), and (**c**) cyclic voltammetry curves of the glassy carbon electrode (GCE) modified with Cu–metal organic frameworks (Cu-MOFs) in the absence (dotted line) and presence (solid line) of 2 mM H_2_O_2_ (Reprinted with permission from [194] Copyright (2016) Royal Society of Chemistry).

Copper nanoparticles (CuNPs) have also gained increased attention due to their highly efficient catalytic properties toward H_2_O_2_ with a low applied potential, which can reduce interference signals from other existing electroactive species [189,190]. Although CuNPs have proven to be effective for the detection of H_2_O_2_, their aggregation has frequently resulted in a reduction of the sensor sensitivity. To mitigate this problem, some groups focused their efforts on the development of methods to prevent the aggregation of CuNPs and Cu_2_O on electrode surfaces [191], as these have shown poor conductivity and stability at ambient conditions [191,192,193]. For example, Wu et al. [194] developed a metal organic framework (MOF)-derived Cu_2_O/CuO@rGO composite by thermally treating a mixture of MOF-118 and GO nanosheets under nitrogen atmosphere at 400, 600, and 800 °C. They concluded that the reduced GO nanosheets in MOFs effectively prevented the aggregation of metal oxide NPs, as shown in Figure 10a, leading to high catalytic performance toward the oxidation of H_2_O_2_ (Figure 10b). The detection of H_2_O_2_ by the Cu-MOF/GO-modified electrode is represented by the presence of an oxidation peak in cyclic voltammetry in Figure 10c. This electrochemical sensor showed a large specific surface area with high conductivity, and a transport pathway that facilitated the electron transfer between the redox metal oxide and the electrode surface. The high dispersion of metal oxide NPs on the electrode surface led to superior detection of H_2_O_2_ with an LOD of 0.71 μM. Golsheikh et al. [195] also used Cu-based MOFs incorporated with GO for H_2_O_2_ detection, obtaining an LOD of 0.44 μM, which is similar to that of Wu et al. [194]. Another interesting approach was reported by Bui et al. [196], who came up with the idea of using alternating current (AC) plasma deposition to fabricate catalytic electrochemical sensors for the detection of H_2_O_2_. Among the different metal oxides explored, CuO NPs provided good sensitivity, selectivity, stability, low LOD (0.6 μM), and a fast sensor response.

Although monometallic electrochemical sensors have proven to be capable of effectively measuring the presence of H_2_O_2_, it is still necessary to improve their conductivity, electrocatalytic activity, and stability to achieve higher sensitivity, selectivity, and repeatability [197]. Thus, in recent years, bimetallic NPs have gained attraction due to their exceptional optical, catalytic, and electronic properties. The use of bimetallic NPs has greatly helped the development of electrochemical-based sensors for the detection of H_2_O_2_ in biological samples, such as human cancer cells [198], human serums, and human urine [197]. Therefore, bimetallic NPs have the potential to enhance the overall sensor performance through higher conductivity, greater selectivity, and less sensor deactivation, making them more suitable for biological systems [199,200,201].

It has been proven that electrochemical activity and sensitivity significantly improve with the use of bimetallic NPs [202]. For example, different combinations of more than one transition metal, such as Au with Ag [203,204,205], Co [205,206], Pt [201,207,208], Pd [209], Cu [210,211], Fe_3_O_4_ [212,213], or Fe_2_O_3_ [212,213], have been intensively investigated and have led to successful development of electrochemical sensors. For instance, Zhao et al. [204] developed Ag-Au bimetallic NPs supported on reduced GO with alginate as a stabilizer and reductant. The combination of Ag and Au was chosen because of its high catalytic activity, biocompatibility, and ease of synthesis. The experimental results evidenced that Ag-Au bimetallic NPs provide a higher number of catalytic active sites than monometallic ones. Moreover, the electrochemical sensor using Ag-AuNPs showed a better performance in terms of H_2_O_2_ detection with a LOD of 0.57 µM, which is much lower than that obtained with most Ag-based sensors.

**Figure 11:**
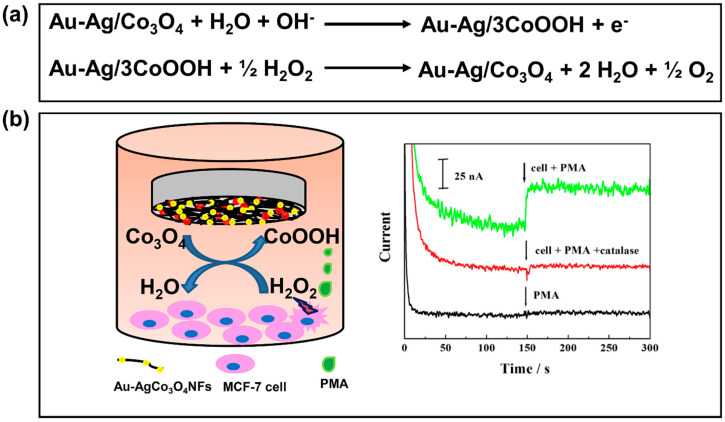
(**a**) Proposed redox mechanism of Au-Ag/Co_3_O_4_ and Au-Ag/CoOOH with hydrogen peroxide (H_2_O_2_) and (**b**) experimental design of a glassy carbon electrode with bimetallic Au-Ag/Co_3_O_4_ nanofibers (Au-Ag/Co_3_O_4_ NFs/GCE) for H_2_O_2_ detection in MCF-7 cells (breast cancer cells) stimulated with phorbol 12-myristate 13-acetate (PMA) and amperometric responses with the Au-Ag/Co_3_O_4_ NFs/GCE in the absence and presence of MCF-7 cells and with the addition of 10 mg·mL^−1^ of PMA and 200 U·mL^−1^ of catalase (Reprinted with permission from [205] Copyright (2018) Elsevier Publishing).

In addition to the advantages described above, Zhang et al. [205] discovered another benefit of bimetallic NPs by integrating them with nanofibers. When they deposited Ag-Au bimetallic NPs on Co_3_O_4_ nanofibers (Au-Ag/Co_3_O_4_ NFs), they found that this material generated the highest Faradic current from the redox reaction with H_2_O_2_ compared to Au/Co_3_O_4_ NFs and Ag/Co_3_O_4_ NFs. The proposed mechanism is shown in Figure 11a. The excellent sensor response toward H_2_O_2_ was attributed to the synergistic effect between Au and Ag and the large surface-to-volume ratio of Co_3_O_4_ NFs. Remarkably, this sensor was successfully used in human breast cancer cells, with an increase in the current response to H_2_O_2_, as shown in Figure 11b. Thus, it should be highlighted that this electrochemical sensor can be used to monitor steady-state concentrations of H_2_O_2_ in human breast cancer cells.

Several other research groups have employed the idea of combining more than one metal to fabricate electrochemical sensors for the detection of H_2_O_2_ in living cells [214,215]. For example, Mani et al. [216] obtained an extraordinarily low LOD in the nanomolar range by modifying nickel cobalt sulfide/cobalt sulfide nanostructure arrays (NiCo_2_S_4_@CoS_2_ NAs) with a carbon cloth (CC). The NiCo_2_S_4_@CoS_2_ NAs provided a 3D hierarchical network with a highly porous structure and excellent conductive properties. Furthermore, the stability of this sensor was greater than most modified electrodes reported in the literature. Likewise, the synergistic effect of the metal compounds was also thought to contribute to the superior electrocatalytic activity toward H_2_O_2_ reduction. Remarkably, the sensor was able to detect the presence of H_2_O_2_ in a phosphate buffer solution with a LOD as low as 2 nM. Additionally, they used this sensor for real-time detection of H_2_O_2_ released by mammalian cells, this being of 0.97 pM of H_2_O_2_, which is consistent with the results obtained with a standard fluorometric H_2_O_2_ assay kit. Importantly, this work proved the possibility of using a combination of metal compounds to modify an electrochemical sensor for the detection of sub-picomole levels of H_2_O_2_ in living cells with high accuracy. Other reports in the literature also combined different metal compounds for detection of H_2_O_2_, but they worked in the nanomolar range [214,217,218].

However, just like in the case of monometallic electrochemical sensors, the bimetallic ones can also suffer from the aggregation that reduces the sensor efficiency. To overcome this challenge, Sakthivel et al. [219] successfully prepared non-aggregated NiFe_2_O_4_ nanosheets to modify an electrode for the detection of H_2_O_2_. As the aggregation was prevented, the modified sensor showed a larger surface area and better conductivity than other aggregated ones, leading to a significant improvement in the LOD to 12.4 pM. The sensor also demonstrated great performance in the detection of extremely low concentrations of H_2_O_2_ in rat brain and human blood serum samples.

From our observations, we can conclude that bimetallic electrochemical sensors are more widely used for the detection of H_2_O_2_ in live cells than monometallic ones, due to their higher catalytic activity. However, the overall sensitivity and selectivity of the sensors still need to be further improved for the detection of picomolar concentrations in a wider linear range for its use as the standard method for the detection of H_2_O_2_ in live cells.

## 4. Hydroxyl Radical (•OH) Detection

### 4.1. Introduction to Hydroxyl Radical (•OH) Detection

Hydroxyl radicals (•OH) are considered the most powerful and potentially dangerous reactive oxygen species (ROS) produced in biological systems due to their ability to react with any cellular materials and macromolecules [220]. As mentioned in the hydrogen peroxide (H_2_O_2_) discussion, •OH are produced in a two-step reaction involving the presence of H_2_O_2_ and transition metals. In the first step, $O_{2}^{•-}$ allows for the reduction of ferric into ferrous ions [221]:

$${\mathrm{Fe}}^{3+}+O_{2}^{•-}\to {\mathrm{Fe}}^{2+}+O_{2}$$

Then, the second step is the Fenton reaction, which involves H_2_O_2_:

$${\mathrm{Fe}}^{2+}+H_{2}O_{2}\to {\mathrm{Fe}}^{3+}+{\mathrm{OH}}^{-}+•\mathrm{OH}$$

As in the case of $O_{2}^{•-}$ and H_2_O_2_, •OH can cause extensive damage to proteins, DNA, and lipids, leading to the disturbance of the normal cellular functions. However, due to the lack of enzymatically catalyzed detoxification mechanisms to scavenge •OH within physiological processes, the overproduction of •OH is much more probable, increasing the likelihood of cell death [222]. This type of harm is the result of protein damage due to the oxidation of the thiol group of cysteine, which, in turn, negatively affects the overall enzymatic activity. Additionally, through lipid peroxidation (LPO), normal membrane structures can be disrupted through the oxidation of polyunsaturated fats by •OH. LPO is especially concerning due to the formation of unstable lipid radicals that lead to repeated cycles of the formation of fatty acid peroxyl radicals, which are believed to propagate cell injury and cause additional damage to cell membranes (e.g., liver injury) [223,224]. Along with liver diseases, the presence of •OH has been linked to diabetes [225,226], various cancers [227,228], and neurodegenerative diseases, such as Alzheimer’s and Parkinson’s [226,229]. As living organisms have complex antioxidant systems to prevent ROS-induced oxidative damage, researchers have focused their attention on measuring antioxidants within a body and the total antioxidant capacity in biological fluids. Thus, much research around •OH is regarding the use of antioxidants and free-radical scavengers and the study of whether the consequences of oxidative stress can be counteracted and/or prevented by these methods.

Common techniques for •OH detection include spin traps with electron paramagnetic resonance (EPR) spectroscopy [230,231], fluorescence [232,233], chemiluminescence [234,235], and electrochemical methods [236]. Similar to $O_{2}^{•-}$ and H_2_O_2_, the detection of •OH is challenging due to its high chemical reactivity, extremely short lifetime (about 10^−9^ s in biological systems), and the difficulty of detecting this species and studying its relationship to DNA damage within model systems. So far, the fluorescence method has shown to be promising due to high selectivity, sensitivity, and the ability to detect •OH in living cells through real-time analysis.

### 4.2. Fluorescence Method for Hydroxyl Radical (•OH) Detection

While fluorescence imaging offers many advantages as a detection method, there are several limitations when developing successful probes for real-time detection. These include instrument sensitivity, source light fluctuations, temperature, concentration variations, and environmental factors [237]. Additionally, as hydroxyl radicals (•OH) are present at low concentrations and have a short lifetime, it can be extremely difficult to properly detect their presence in vivo. Imaging at the second near-infrared (NIR-II, 1000–1700 nm) window has been a growing area of interest due to its several advantages, such as reduced absorption by surrounding tissues, lessened light scattering, and autofluorescence from biosamples [238,239,240].

**Figure 12:**
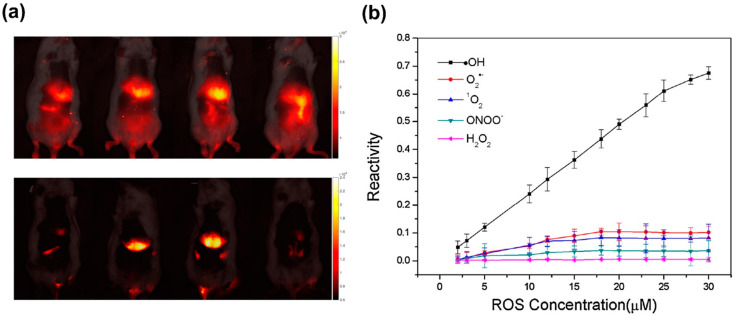
(**a**) Second near-infrared (NIR-II) and NIR-IIa fluorescence images of mouse organs (Reprinted with permission from [241] Copyright (2019) ACS Publications). (**b**) Sensor selectivity of the binary nanoprobe to different reactive oxygen species (ROS) concentrations (Reprinted with permission from [237] Copyright (2019) Elsevier Publishing).

Although NIR-II imaging has shown potential for high selectivity and sensitivity, some issues have led to difficulties with the probe design, such as the fact that limited molecules can absorb and emit within the desired wavelength and the limited choices of quenchers. However, by observing the fluorescent intensity by breaking the conjugated system and structure of an organic fluorophore, Feng et al. [241] successfully developed an NIR-II fluorescent probe that displayed sensor sensitivity to •OH both in vitro and in vivo, confirming the practicality of the probe for non-invasive monitoring of •OH. To fabricate a fluorescent probe with a high selectivity for •OH, they chose cyanine molecules as a sensing element. The selective reaction between •OH and cyanine molecules occurred at the C−N bonding, resulting in fluorescence emission at 1044 nm. By exhibiting excellent selectivity and sensitivity with a limit of detection (LOD) of 0.5 nM, it was possible to obtain clear imaging of the changing levels of •OH within mouse organs, as shown in Figure 12a.

A second solution to the common challenges associated with fluorescence detection for •OH was proposed by Cong et al. [237], who employed a ratiometric fluorescence detection method. This study involved the development of cyanide-based nano-Group of Uniform Materials Based on Organic Salts (nanoGUMBOS) to be used as a fluorescent probe for •OH detection. Remarkably, their probe displayed a higher sensitivity and selectivity toward •OH compared to other reactive oxygen species (ROS) (Figure 12b), and it was able to overcome the challenge associated with the short lifetime of •OH, enabling the detection of •OH in human breast cancer cells.

**Figure 13:**
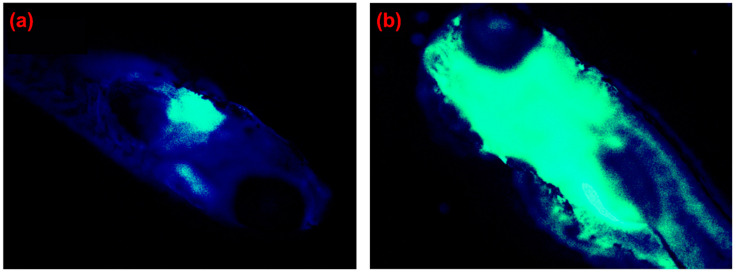
Fluorescence imaging of zebrafish incubated with a fluorescent probe (**a**) before hydroxyl radical (•OH) generation and (**b**) after •OH generation (Reprinted with permission from [242] Copyright (2019) Elsevier Publishing).

When developing new fluorescence detection methods, the importance of detection within living cells remains a top priority. Although there has been much development around •OH-detecting fluorescent probes, there is still a lack of advancement in the development of probes targeted to specific organelles. Because ROS, including •OH, are primarily produced in mitochondria, probing a specific organelle with high •OH selectivity is essential for the use of •OH as a biomarker, serving as a diagnostic tool at the beginning of many oxidative-stress-related diseases. For example, Yuan et al. [242] investigated the use of a xanthene derivative azo dye as a quenching and recognition group of •OH. This work led to the development of a xanthene fluorescent probe with a mitochondria-targeted function. Their developed probe consisted of two essential groups: a lipophilic cation and an azo group. For detecting •OH, the azo group acted as a recognizing-group of •OH. Specifically, •OH would selectively attack and cleave an azo bond in the probe, resulting in fluorescence emission at 550 nm. This novel probe proved to be nontoxic and was able to detect •OH in vitro and in vivo at the sub-organelle level by providing imaging of •OH in both mitochondria and living zebrafish, as shown in Figure 13.

### 4.3. Electrochemical Methods for Hydroxyl Radical (•OH) Detection

While there have been advancements in the development of fluorescent probes for hydroxyl radical (•OH) detection, electrochemical sensing has arisen as an attractive technique due to its fast response, high selectivity, sensitivity, economic feasibility, and simplicity of use. Electrochemical sensing technologies for the detection of •OH are categorized as organic and inorganic approaches. Organic-based electrochemical sensors use components such as DNA [243,244], conductive polymers [245,246,247,248], and organic molecules [249,250]. On the other hand, inorganic electrochemical methods rely on metal oxide nanoparticles [251,252,253] and carbon-based materials [254,255]. Among them, the most common electrochemical sensors for the detection of •OH are those in which organic elements are immobilized on an electrode surface to act as the sensing element toward •OH.

The idea of using DNA as a sensing element emerged from the fact that DNA is easily and directly damaged by the oxidation reaction with •OH [256,257]. For example, Wu et al. [243] demonstrated the successful detection of •OH with DNA-functionalized gold nanoparticles (AuNPs) on the surface of a gold electrode. DNA was combined with AuNPs to amplify the sensor signal response, which was believed to improve the sensitivity and selectivity of the electrochemical sensor toward •OH. By combining DNA with AuNPs, this group was able to detect •OH in a wide linear range from 5 to 10 mM with a limit of detection (LOD) of 3 µM. Consequently, there has been continuous focused attention on the development of electrochemical DNA biosensors for increased sensitivity toward •OH detection. For instance, Huang et al. [244] further enhanced the sensitivity and LOD of electrochemical probes by constructing a biosensor using DNA and 6-mercaptohexanol (MCH) with nitrogen-doped porous carbon materials and AuNPs as signal amplifiers. They demonstrated that the degree of DNA oxidative damage was related to the concentration of •OH, and they obtained good sensitivity toward •OH, with a LOD as low as 25 µM.

Abdel-Hamid et al. [258] were also interested in using a strategy that involved the use of DNA as a sensing element for the detection of •OH. As antioxidants are known to act as reducing agents for protection against free-radical damage, their study was driven by the desire to identify a natural antioxidant. Previous studies had reported that caffeic acid (CAF) protects against •OH formation by scavenging free radicals, which could protect DNA from degradation [259]. Therefore, this group modified a glassy carbon electrode with a double-stranded DNA (dsDNA) layer immobilized on multi-walled carbon nanotubes to investigate the interaction with CAF and evaluate antioxidant properties. During this study, the researchers detected the oxidative damage caused to DNA by •OH, and it was observed that, upon addition of CAF, the dsDNA was protected due to its •OH-scavenging properties.

**Figure 14:**
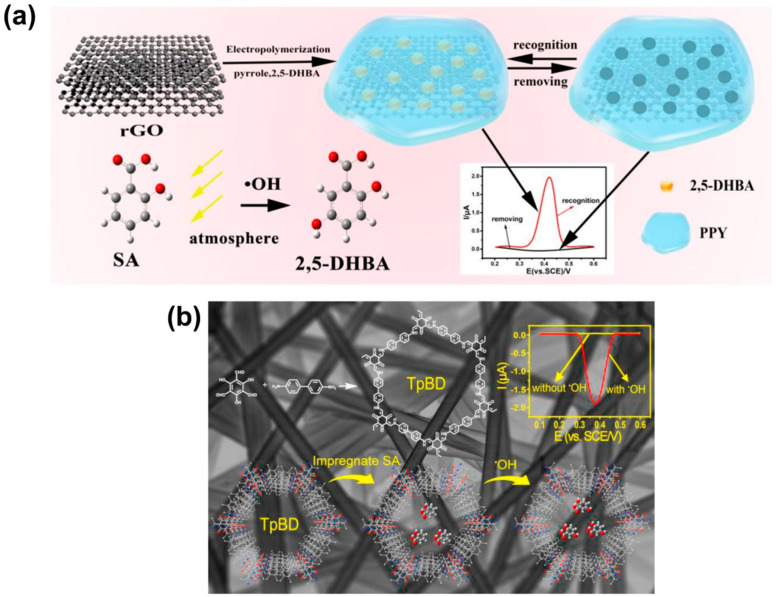
(**a**) Sensor preparation and working mechanism of a molecularly imprinted polymer-based electrochemical sensor for the detection of hydroxyl radical (•OH) (Reprinted with permission from [246] Copyright (2019) Royal Society of Chemistry). (**b**) Modification of a carbon fiber paper for the detection of •OH (Reprinted with permission from [262] Copyright (2019) Elsevier Publishing).

Even though these DNA-based biosensors are easy to prepare without using complicated procedures and toxic chemicals, they are forced to operate in limited situations due to the degradation of DNA in severe and extreme environments. Another approach to the development of organic-based electrochemical sensors is the combination of molecularly imprinted polymers (MIPs) with electrochemistry. For example, Huang et al. [246] developed an electrochemical sensor for the detection of •OH by measuring the concentration of the electroactive product 2,5-dihydroxybenzoic acid (2,5-DHBA) obtained from the reaction between salicylic acid (SA) and •OH. To do so, 2,5-DHBA was imprinted by using an electrochemical method in the presence of reduced graphene oxide (rGO) and pyrrole as a functional monomer. This composite resulted in an electrode surface containing cavities with the shape and size of 2,5-DHBA for selective detection. The synthesis method and working mechanism of the MIP-based electrochemical sensor for the detection of •OH is shown in Figure 14a. The molecular imprinting technique was used to provide the sensor with selectivity, and rGO was used to increase the number of active sites for 2,5-DHBA to be imprinted. Remarkably, the researchers found that 2,5-DHBA could selectively rebind on the surface cavities of the MIP/rGO-modified electrochemical sensor and induce a sensor response, which was directly related to the concentration of •OH. Interestingly, the MIP-based electrochemical sensor was able to detect •OH in a linear range from 5.0 × 10^−8^ to 4.5 × 10^−5^ M with a LOD as low as 2.74 nM. Additionally, since the cavities on the surface were designed to have the specific size and shape of 2,5-DHBA, there was no question about the selectivity toward the product obtained from the reaction between SA and •OH.

Although this type of electrochemical sensor is suitable for in vitro •OH detection, further improvements are necessary for its use in complex biological systems. For example, complex environments could contain molecules smaller than the molecularly imprinted cavities, and these could block the cavities on the electrode surface, leading to a significant reduction in the sensor sensitivity and selectivity. The same strategy of using a specific product generated from the reaction between a trapping agent and •OH to determine the concentration of •OH was also investigated by Wang et al. [260]. Another example was provided by Huang et al. [261], who successfully detected •OH in the atmosphere with a material synthesized by the impregnation of SA (trapping agent) on carbon fiber paper (CFP). However, further development was required to improve the sensor sensitivity and reduce the detection time. Then, in 2020, Huang et al. introduced covalent organic frameworks (COFs) on the surface of CFP to increase the amount of impregnated SA, which could lead to increased sensitivity toward •OH [262]. Once the impregnated SA reacted with •OH on the CFP surface, the electrochemical signal of 2,5-dihydroxybenzoic acid (2,5-DHBA) was measured for the indirect determination of •OH. Figure 14b shows the surface of CFP modified with 1,3,5-triformylphloroglucinol (Tp) and benzidine (BD) (COF(TpBD)), and Figure 14a shows its preparation and application to the detection of •OH. The addition of (COF(TpBD)) to CFP resulted in an increased amount of the trapping agent and a significant reduction in the LOD toward •OH (6.9 × 10^−15^ mol/L or 0.0069 pM) when compared to the CFP without surface modification from their previous work. This group introduced the idea of combining well-known organic molecules, such as SA, with an advanced surface modification technique to develop electrochemical sensors with high sensitivity and selectivity toward •OH. However, by looking at the recent literature (2016 to 2020), it can be concluded that many researchers have slowly departed from using DNA as a sensing element compared to other organic molecules. While the reasoning behind this is not clear, it is likely due to the vulnerability of DNA in severe and extreme environments.

**Figure 15:**
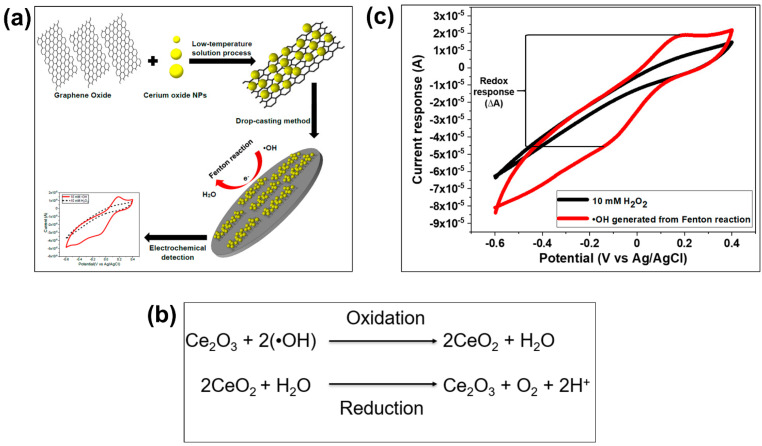
(**a**) Development of a cerium oxide nanoparticle/graphene oxide (CeNP/GO)-based electrochemical sensor, (**b**) redox reaction between CeNPs and hydroxyl radical (•OH), and (**c**) sensor redox response in the presence of •OH (Reprinted with permission from [252] Copyright (2020) Elsevier Publishing).

As mentioned above, the use of organic molecules as a sensing element in electrochemical sensors poses several challenges that might be overcome by the use of inorganic materials. For example, Duanghathaipornsuk et al. [236,252] developed a cerium oxide/graphene oxide composite (CeNP/GO composite)-based electrochemical sensor for the detection of •OH (Figure 15a). Cerium oxide nanoparticles (CeNPs) were chosen as a sensing element due to their outstanding ability to scavenge •OH via the redox reaction shown in Figure 15b. Even though CeNPs have dual oxidation states (i.e., Ce^3+^ and Ce^4+^), Ce^3+^ are considered the active sites for the detection of •OH (Figure 15b). To overcome the poor conductivity of CeNPs, they were combined with graphene oxide (GO) due to its intrinsic high conductivity and large surface area. Remarkably, these CeNP/GO composites presented both high sensitivity and selectivity toward •OH. Leveraging on that result, the researchers also investigated the effects of the size and content of CeNPs on the sensor redox response toward •OH (Figure 15c). These CeNP/GO composite-based electrochemical sensors showed a linear relationship between the sensor redox response and the concentration of •OH. Moreover, they found that the size and content of CeNPs had a great impact on the sensor sensitivity toward •OH. For example, 50 wt% of 16 nm CeNPs in the CeNP/GO composite showed a sensor sensitivity of 470 µM toward •OH as compared to 50 wt% of 8 nm CeNPs (85 µM) due to the higher content of Ce^3+^ in the smaller CeNPs. Additionally, it was found that the composite with a weight ratio of 50:50 CeNPs:GO provided the highest sensor redox response to •OH, which was attributed to a compromise between good conductivity (GO) and reduced agglomeration of CeNPs (higher concentration of Ce^3+^ sites). As a result, this work paved the way for the use of inorganic elements for the detection of •OH.

**Figure 16:**
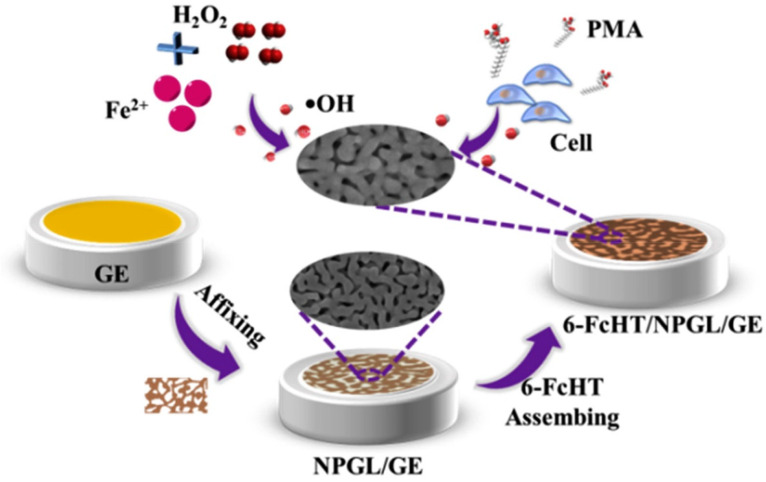
Schematic of the preparation of 6-ferrocenyl hexanethiol self-assembled nanoporous gold layer–modified gold electrode (6-FcHT/NPGL/GE) electrochemical sensor for the detection of hydroxyl radical (•OH) (Reprinted with permission from [250] Copyright (2020) Elsevier Publishing).

Although organic and inorganic electrochemical methods have shown promising results in the detection of •OH, researchers have also explored the combination of both organic and inorganic elements in an attempt to improve the sensitivity and selectivity of the sensors, while addressing common obstacles associated with the electrochemical method. For example, in studies focused on inorganic electrochemical detection, selectivity turns out to be one of the key issues due to the difficulty of specifically recognizing the target molecule. Sensitivity has been reported to be another drawback due to the limited surface area of the bare electrode. To overcome this challenge, AuNPs have been commonly used to enhance the sensitivity by expanding the surface area of the electrode, thus improving the electron transfer rate. While AuNPs have provided substantial improvement, they are considered to have two-dimensional material properties, which still limits the surface area. Xu et al. [250] studied the use of a nanoporous gold layer (NPGL) with a 3D interconnected structure to increase the pore size and enhance the overall surface area. By providing more active sites for •OH capture, the NPGL can significantly enhance the sensor sensitivity when modified with 6-(ferrocenyl) hexanethiol (6-FcHT) (Figure 16). The performance of this sensor was examined by electrochemical impedance spectroscopy (EIS), cyclic voltammetry (CV), and square-wave voltammetry (SWV), demonstrating that the NPGL has an important effect. As a matter of fact, when the 6-FcHT-modified gold electrode (6-FcHT/GE) was directly used to detect •OH, the sensitivity recorded was of 0.0305 mA nM^−1^, with a LOD of 0.133 nM; however, after NPGL modification, the sensitivity increased to 0.1364 mA nM^−1^, with a remarkable LOD of 0.316 pM. This electrochemical sensor was also tested in vitro in a biological system using human liver cancer line (HepG2) culture solutions, and it proved to be able to detect increased concentrations of •OH in treated HepG2 cells. Additionally, as expected, the researchers also observed that the electrical current significantly decreased upon the addition of antioxidants, such as ascorbic acid, uric acid, and glutathione.

**Figure 17:**
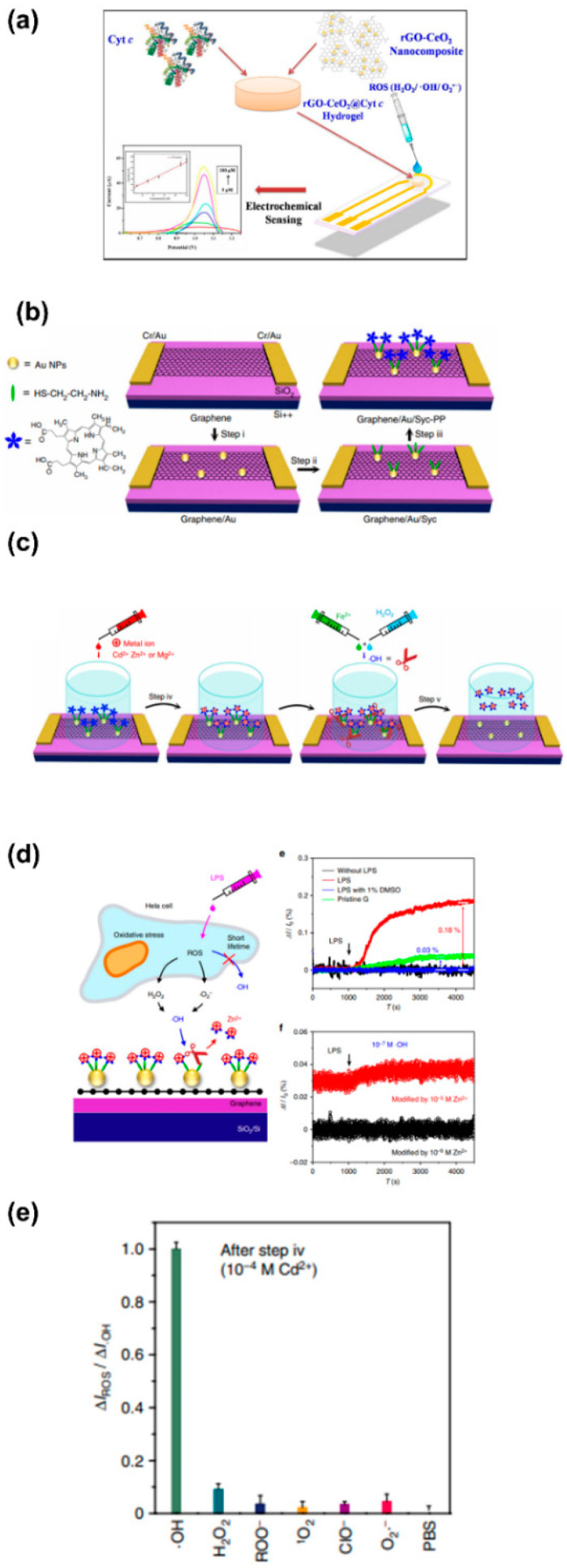
(**a**) Sensor development with a reduced graphene oxide-cerium oxide nanocomposite and cytochrome c alginate-polyacrylamide hydrogel-sensing platform (rGO-CeO_2_@Cyt-c hydrogel/SPE) (Reprinted with permission from [40] Copyright (2020) Royal Society of Chemistry). (**b**) Preparation of a graphene/gold nanoparticle-based field-effect transistor immobilized by a self-assembly monolayer of cysteamine and protoporphyrin IX (graphene/Au/Cys-PP/FET)-based electrochemical sensor, (**c**) detection of hydroxyl radical (•OH) via the inner-cutting strategy, (**d**) detection of released •OH from HeLa cells (immortal cell line), and (**e**) sensor selectivity of graphene/Au/Cys-PP/FET toward •OH (Reprinted with permission from [269] Copyright (2019) Springer Nature).

Other studies in the literature have reported that immobilization of cytochrome-c (Cyt-c), a redox protein, on solid surfaces or nanomaterials, such as graphene [146], carbon nanotubes [263,264], or metal and metal oxide NPs, can significantly enhance the electrochemical performance [104,265,266,267]. While Cyt-c is commonly involved in catalyzing the reduction of reactive oxygen species (ROS), it shows poor activity when loaded on a bare electrode surface [268]. Consequently, recent studies have focused on the incorporation of hydrogels for the detection of ROS. Hydrogels have many industrial applications due to their porous structure and large surface area, mechanical stability, and good biocompatibility. Additionally, hydrogels and biomaterials have a great synergistic effect, which can contribute to the detection of biological analytes via the electrochemical method. As an example, Kumar et al. [40] studied how to further enhance the performance of hydrogels through the integration of nanomaterials and biomolecules that are responsive to ROS. To do so, they combined a reduced graphene oxide–cerium oxide nanocomposite (rGO-CeO_2_) and Cyt-c to synthesize a multicomponent alginate–polyacrylamide hydrogel-sensing platform (rGO-CeO_2_@Cyt-c hydrogel/SPE) (Figure 17a). This hydrogel-based assembly provided a large surface area for the incorporation of Cyt-c, and as previous studies suggested that •OH contributes to the depolymerization of hydrogels, rGO-CeO_2_ was integrated into the sensor configuration as a protective agent to prevent the degradation. Furthermore, a hydrated environment can promote the biological functions, increase the conductivity, enhance the sensor signal, and help to resist fouling. Interestingly, the rGO-CeO_2_@Cyt-c hydrogel/SPE sensor exhibited a LOD as low as 0.229 µM, showing the potential of hybrid hydrogels for the real-time detection of ROS, such as •OH, in vitro and in vivo.

A year sooner, Wang et al. [269] used the same concept of combining organic and inorganic elements for the detection of •OH and obtained a remarkable LOD of 0.001 μM. This group developed a graphene/gold nanoparticle-based field-effect transistor (FET), which was immobilized by a self-assembly monolayer of cysteamine and protoporphyrin IX (PP) as a graphene/Au/Cys-PP/FET composite (Figure 17b). Additionally, and to use it for the detection of •OH, they doped the sensor surface with metal ions (Cd^2+^, Zn^2+^, or Mg^2+^) (Figure 17c). Even though the addition of metal ions made the sensor preparation more complex, it helped to enhance the sensor sensitivity toward •OH. The graphene/Au/Cys-PP/FET used the inner-cutting strategy, which is considered an indirect label-free detection method. This approach is based on the selective cut of the self-assembly monolayer of Cys-PP-metal ions by •OH out of the AuNPs on an electrode surface, which leads to the release of metal ions that are used as the sensor response toward •OH, as shown in Figure 17c. Due to the high selectivity and the ultrafast reaction between •OH and cysteamine [270], this sensor was able to detect concentrations of •OH as low as 1 nM. Remarkably, the researchers were also able to detect released •OH in HeLa cells (immortal cell line) within a concentration range of 10^−7^ M using the graphene/Au/Cys-PP/FET (Figure 17d). This combination of cysteamine and AuNPs also provided them with an outstanding sensor selectivity, as shown in Figure 17e.

Other researchers, such as Ding et al. [271], recognized the great challenge of detecting intracellular •OH and, therefore, investigated the ability of in situ monitoring of •OH in living cells at the subcellular level. Even though it is known that oxidative damage can lead to the development of diseases, such as Alzheimer’s, the lack of information about intracellular •OH levels creates a significant roadblock to a further understanding of how these diseases can be effectively treated. Very recently, Ding et al. [271] developed a tungsten nanoelectrode functionalized with 1-hexanethiol (HAT) for the analysis of •OH inside a single living cell without damage. Its performance was evaluated against several ROS, including •OH,$\;O_{2}^{•-}$, H_2_O_2_, ClO^−^, and NO_2_^−^, in a simulated cellular environment. Remarkably, even when those other ROS were at concentrations 10^5^-fold greater than •OH, there was still no response from them, which highlighted the high selectivity of the sensor toward •OH. Later, for intracellular analysis, the researchers stimulated Raw 264.7 (tumor cells induced by leukemia) murine macrophages with β-amyloid peptides (Aβ_1-40_) to cause injury. This is due to the crucial involvement of Aβ as the main component of amyloid plaques found in the brains of those suffering from Alzheimer’s disease. Since Aβ can contribute to the generation of ROS [272,273] and lead to neuronal degeneration, in situ monitoring of •OH production within Aβ-activated microglia could significantly contribute to a better understanding of the progression of Alzheimer’s disease. In addition, the stimulation of Aβ-induced injuries could help to investigate the antioxidant properties of different species. Because of these reasons, Ding et al. [271] decided to treat cells with cordycepin, a species used in Chinese medicine for its anti-inflammatory and neuro-protective properties, in order to study its possible effects on the injury. Then, by monitoring •OH levels in the cytoplasm with cordycepin before and after Aβ_1-40_ stimulation, the team discovered that cordycepin was able to decrease the concentration of •OH and display little cytotoxicity on cells, demonstrating its potential use for cell protection from Aβ-induced cell death.

Heme oxygenase-1 (HO-1) has also received considerable attention for protecting the brain from oxidative injury through the generation of bilirubin, a known antioxidant [274]. In this regard, Bauer et al. [274] showed that cordycepin promoted the expression of HO-1, suggesting that the combination of cordycepin and HO-1 could lead to the direct scavenging of •OH, providing the cells with protection from Alzheimer’s disease-associated oxidative stress injury. The latest findings together with the development of sensors for the detection of •OH at the subcellular level have potential to help the medical community to study the pharmacological effects of antioxidants on •OH-induced cell damage, as well as to find even more effective treatments for ROS-induced diseases.

Since 2016, several researchers have come up with novel ideas to develop fluorescence and electrochemical methods for the detection of •OH, and the sensitivity and selectivity of those sensors have significantly improved to the point of detecting the •OH released from live cells. Even though organic elements provide exceptional selectivity toward •OH, there is still a need to address their vulnerability in severe environments, which can tremendously impact the sensor performance. On the other hand, inorganic-based electrochemical methods show superior performance in terms of sensitivity, which is attributed to the large surface area and conductivity of the inorganic materials (e.g., metal oxides). However, further studies are required to address the poor selectivity of these sensors toward •OH. As a result, the combination of organic and inorganic elements is getting more attraction, as it has potential to enhance both the sensitivity and the selectivity of the developed sensors. Table 1 summarizes the major takeaways from this review.

## 5. Conclusions and Future Perspectives

There are several similarities between fluorescence and electrochemical methods for the detection of reactive oxygen species (ROS), such as the ease of use, excellent sensitivity, and high selectivity. Additionally, the cell permeability of both methods has led to significant results around ROS in live cells and related fatal diseases. The major sensing elements or electrocatalysts for the detection of ROS can be mainly classified into organic and inorganic materials, and their selection should be guided by the detection environment. Recently developed fluorescence and electrochemical methods demonstrate acceptable sensitivity and selectivity for the detection of ROS in live cells; however, the advancements are different depending on the type of ROS. Most technologies for the detection of ROS are focused on the superoxide radical ($O_{2}^{•-}$), both in vitro and in vivo, which is due to the general perception that $O_{2}^{•-}$ is the primary ROS in living cells and that it induces the generation of other ROS. The development of technologies for the detection of hydrogen peroxide (H_2_O_2_), both in vitro and in vivo, has also increased recently. However, in comparison to $O_{2}^{•-}\;$ and H_2_O_2_, there is a serious lack of technologies for the detection of hydroxyl radicals (•OH), especially in live cells. Therefore, it seems necessary to develop sensors with improved sensitivity and selectivity to study the impact of •OH in mammalian cells and related oxidative-stress-induced diseases. Even though the sensitivity and selectivity of both fluorescence and electrochemical methods are similar, the simplicity of the electrochemical method has gained the attention of many researchers. Very importantly, the electrochemical technique also has potential for real-time detection using micro- or even nanoscale electrodes, which can readily lead to the development of real-time in vivo sensors regardless of the type or location of the live cells of interest.

## Figures and Tables

**Table 1:** Advantages, disadvantages, and further studies and required improvements of the different detection technologies for reactive oxygen species (ROS).

	**Reactive Oxygen Species (ROS) Detection**			
Fluorescence Method (make ROS visible in live cells)	**Fluorescence Method for ROS Detection**			
	**Advantages**	**Disadvantages**	**References**	**Further Studies and Improvements**
	A specific generation source of ROS in live cells can be mapped and located.	Biological samples could be damaged due to high-energy light emission, such as photobleaching.	[61,62,71,83]	Novel organic molecules for fluorescent probes are needed to reduce the auto-oxidizing phenomenon.
	Fluorescent probes can be easily modified for different types of ROS.	The concentration of ROS can be over-interpreted via false emission light from surrounding tissues.	[275,276,277]	A better design of fluorescent probes is needed to selectively react with specific ROS.
	It is an easy sensing procedure.	Limited fluorescent molecules can absorb and emit within the desired wavelength.	[65,68]	The water solubility of fluorescent probes needs to be improved.
	It has excellent biocompatibility.	The dynamic concentration of ROS in live cells is hard to evaluate over extended periods of time.	[79,84,278,279,280]	The detection mechanism needs to be studied in detail.
	It is a non-invasive method.		[281,282]	
	**Electrochemical Method for ROS Detection**			
Electrochemical Method (detects ROS by electron exchange)	**Organic Electrochemical Method for ROS Detection**			
	**Advantages**	**Disadvantages**	**References**	**Further Studies and Improvements**
	It is easy to perform without using complicated procedures and toxic chemicals.	Degradation of the sensor can occur in harsh environments.	[47,243,244]	The biological and chemical compatibility of the electrode surface should be improved to be used in live cells.
	It shows high selectivity toward the ROS of interest using a specific enzyme.	The sensor performance depends on the surrounding environment and can be inconsistent under unsuitable conditions.	[89,110,134,243,244]	The design for the detection of ROS at their generation sources in live cells should be enhanced.
	Real-time detection and fast response are possible.	The signals from other coexisting electroactive species can interfere during a high-potential operation.	[47,101,107,134,135]	The protection of biological/organic molecules on the electrode surface against degradation under harsh conditions should be improved.
	Sensor sensitivity can be improved by integrating highly conductive materials.	Biological molecules are costly sensing elements.		A combination of conductive materials with biological/organic molecules should be used to improve the sensor sensitivity and selectivity.
	**Inorganic Electrochemical Method for ROS Detection**			
	**Advantages**	**Disadvantages**	**References**	**Further Studies and Improvements**
	Detection can be performed in severe environments.	The aggregation of inorganic materials can impair the detection performance.	[143,179,198,214]	Additional development of the methods to prevent aggregation of inorganic materials is required.
	Sensor sensitivity and selectivity for individual ROS can be easily adjusted by using different inorganic materials.	The inorganic materials used as sensing elements need to be wisely chosen to selectively detect individual ROS of interest.	[114,252]	Types of inorganic materials as sensing elements should be further investigated to improve the sensor selectivity to specific ROS.
	Real-time detection and fast response are possible.		[116,117,118]	To improve overall sensing elements, methods to control the morphology and distribution of inorganic materials as sensing elements could be further developed.
	It shows low-interference signals from other existing electroactive species.		[115,283,284]	
	It provides long-term stability with excellent reusability.		[47,112]	

## Data Availability

The data presented in this study are available on request from the corresponding author.
